# Supplementary figures and images for: A twin UGUA motif directs the balance between gene isoforms through CFIm and the mTORC1 signaling pathway
Source: eLife. 2023 Sep 4;12:e85036. doi: 10.7554/eLife.85036 (PMC10476966; doi:10.7554/eLife.85036)

Fig. 1D-source data

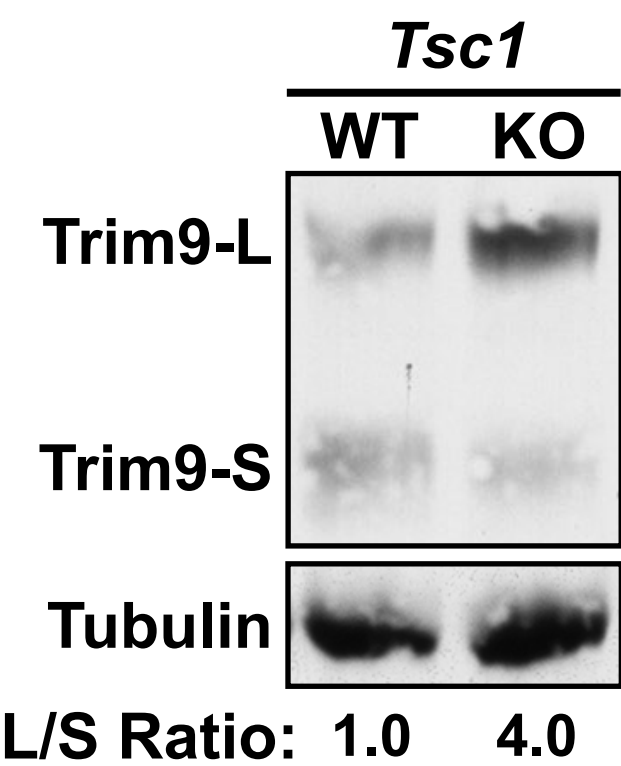

Figure panel

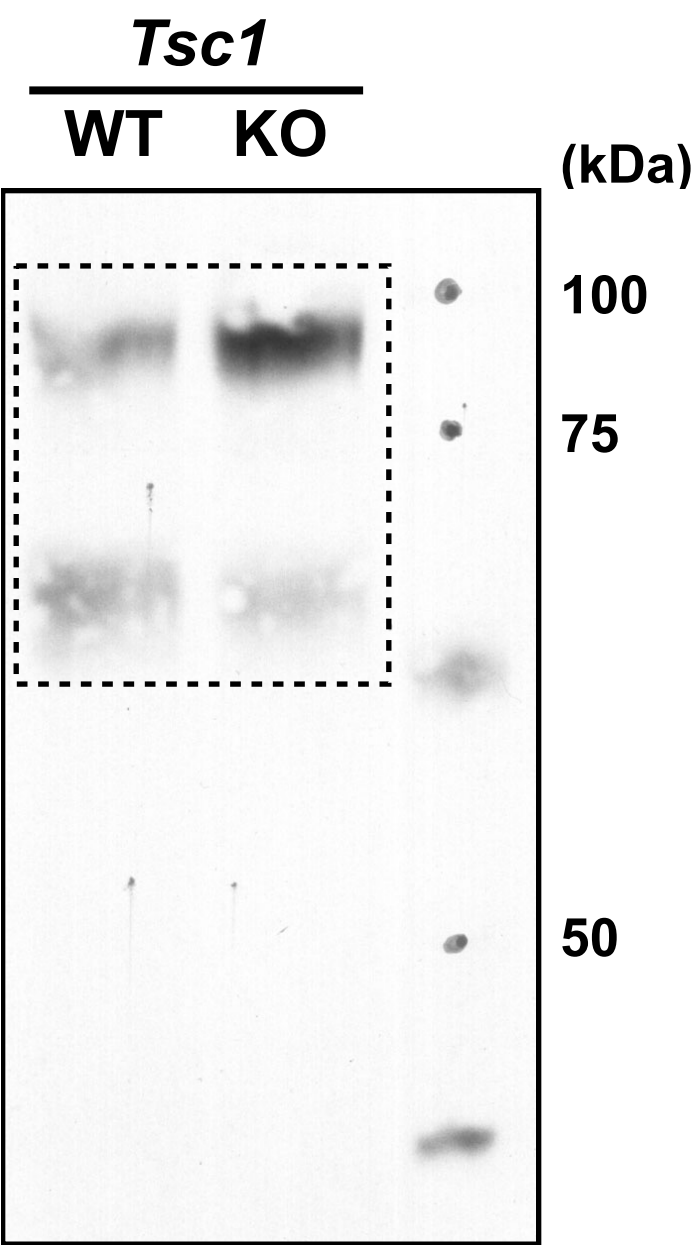

WB: Trim9

Source data 2

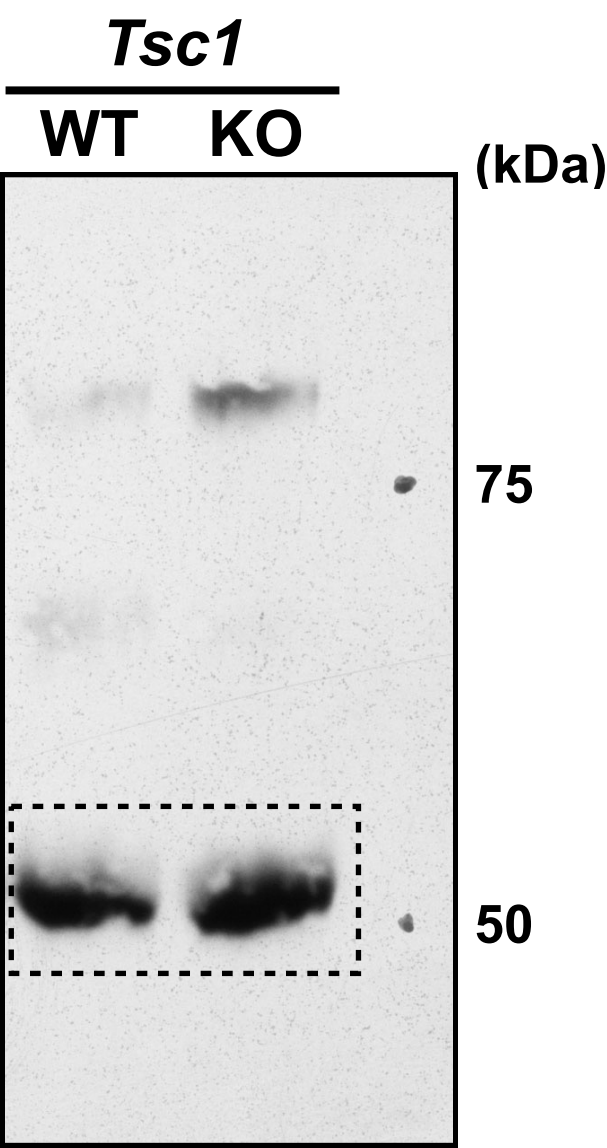

WB: Tubulin (re-blot)

Source data 3

Supplement: Figure 1—source data 1. [file elife-85036-fig1-data1.zip › Figure1_source_data1/Figure1D_sourcedata1.pdf]

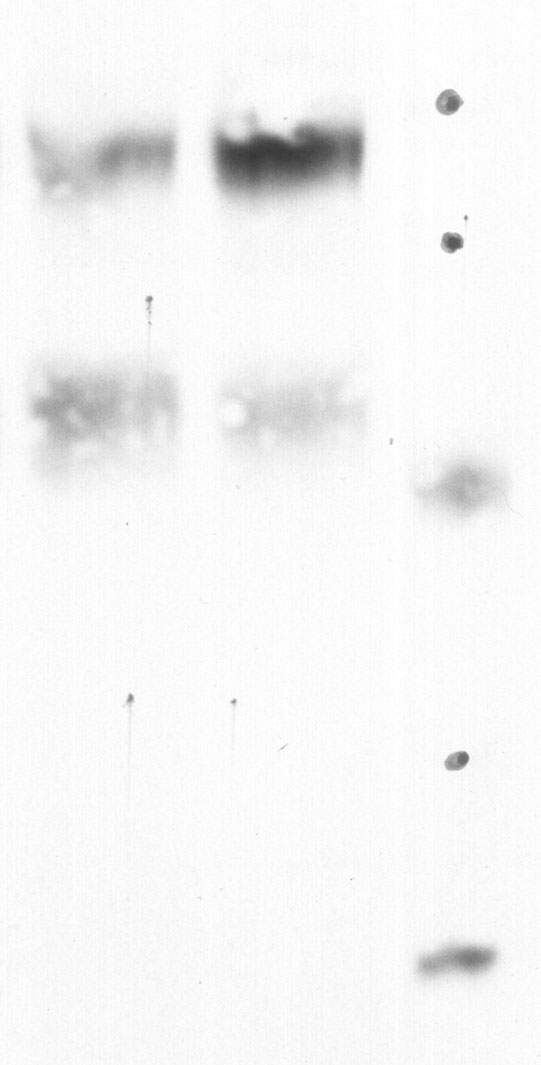

Supplement: Figure 1—source data 1. [file elife-85036-fig1-data1.zip › Figure1_source_data1/Figure1D_sourcedata2.jpg]

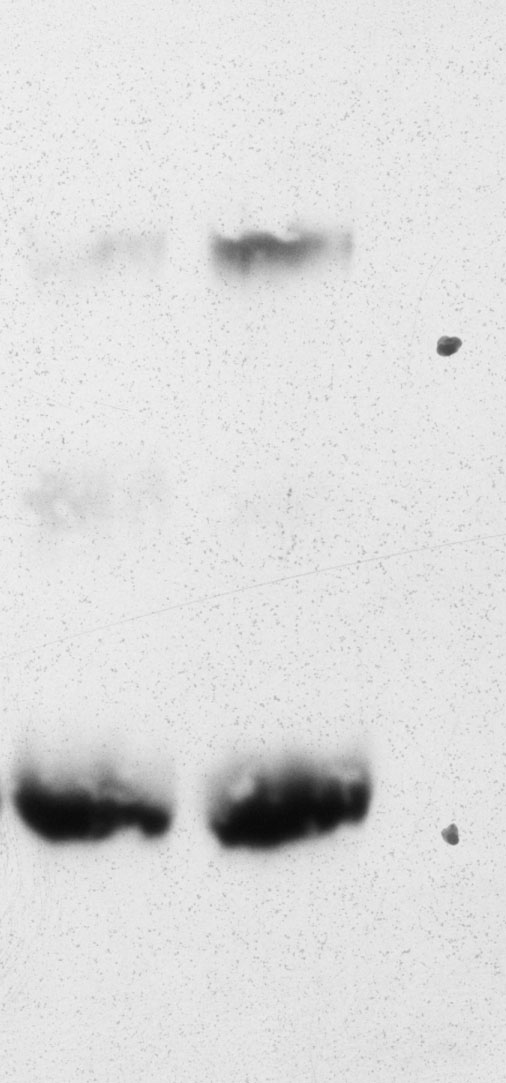

Supplement: Figure 1—source data 1. [file elife-85036-fig1-data1.zip › Figure1_source_data1/Figure1D_sourcedata3.jpg]

Fig. S1B-source data

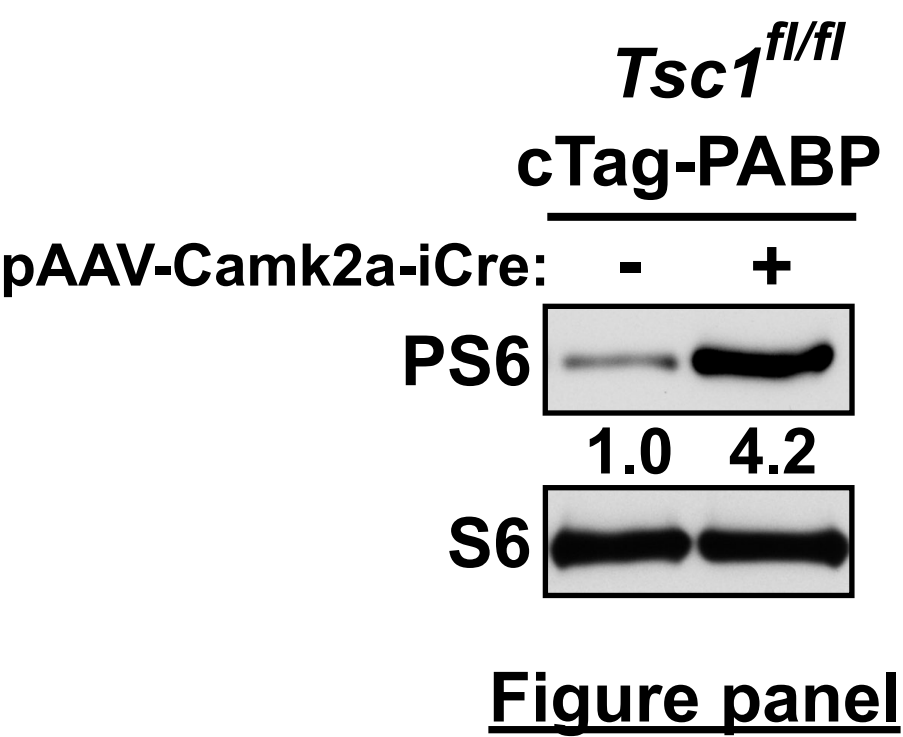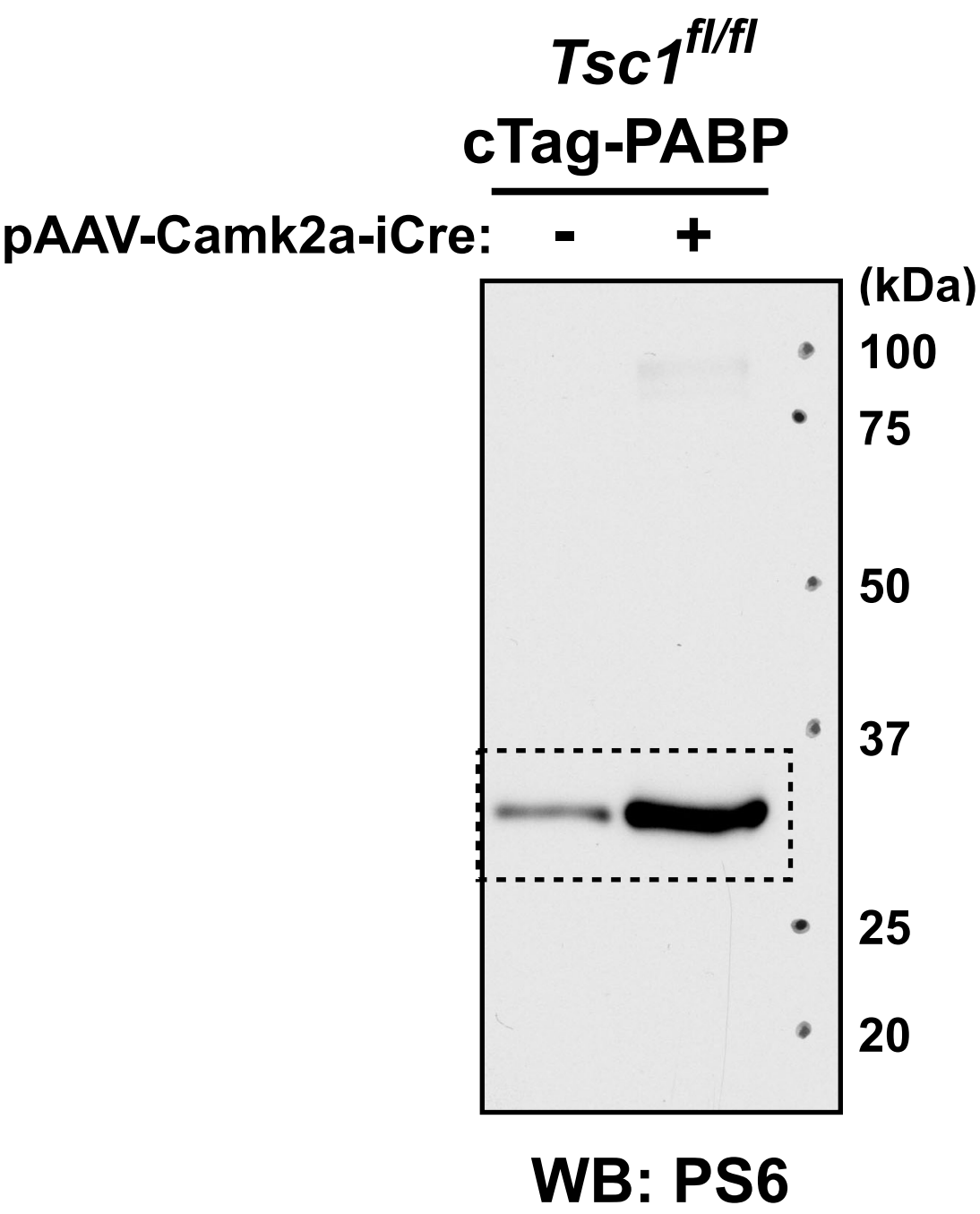

Source data 2

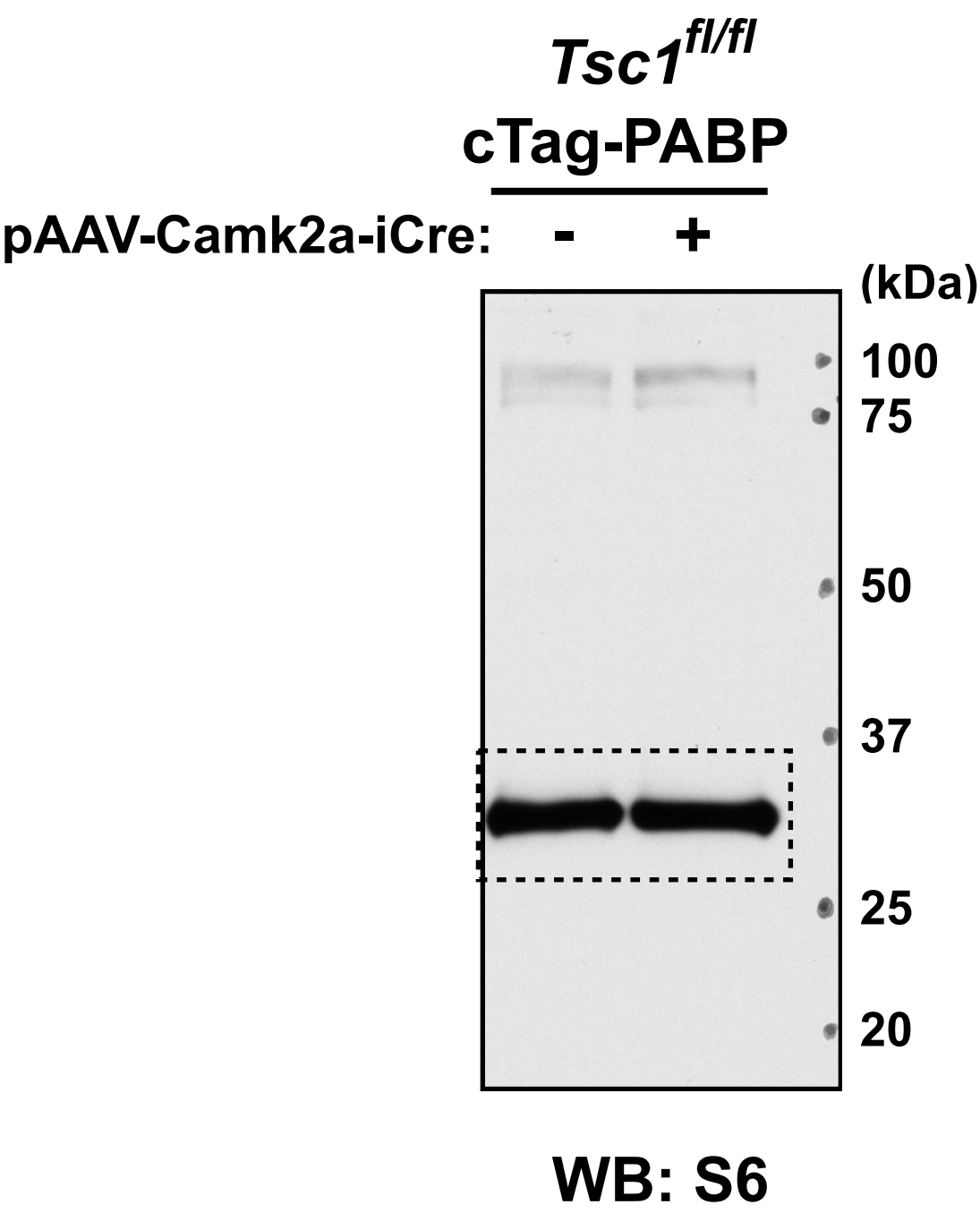

Source data 3

Supplement: Figure 1—figure supplement 1—source data 1. [file elife-85036-fig1-figsupp1-data1.zip › Figure1_supplement1_source_data1/FigureS1B_sourcedata1.pdf]

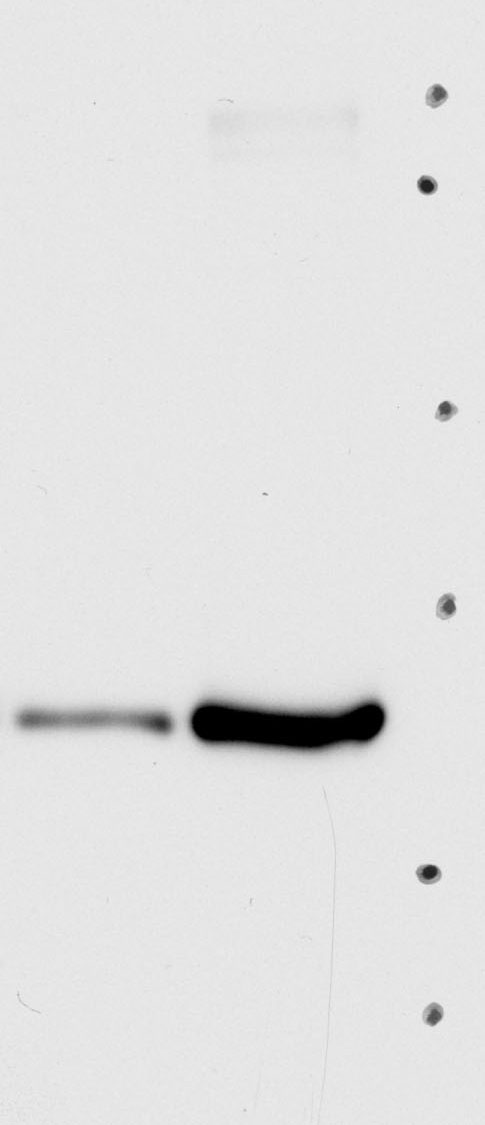

Supplement: Figure 1—figure supplement 1—source data 1. [file elife-85036-fig1-figsupp1-data1.zip › Figure1_supplement1_source_data1/FigureS1B_sourcedata2.jpg]

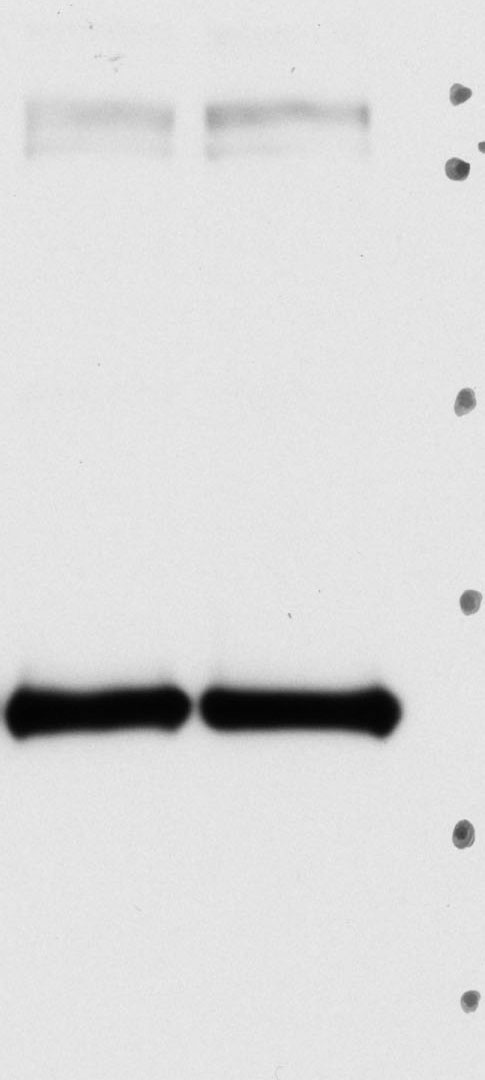

Supplement: Figure 1—figure supplement 1—source data 1. [file elife-85036-fig1-figsupp1-data1.zip › Figure1_supplement1_source_data1/FigureS1B_sourcedata3.jpg]

Fig. S1C-source data

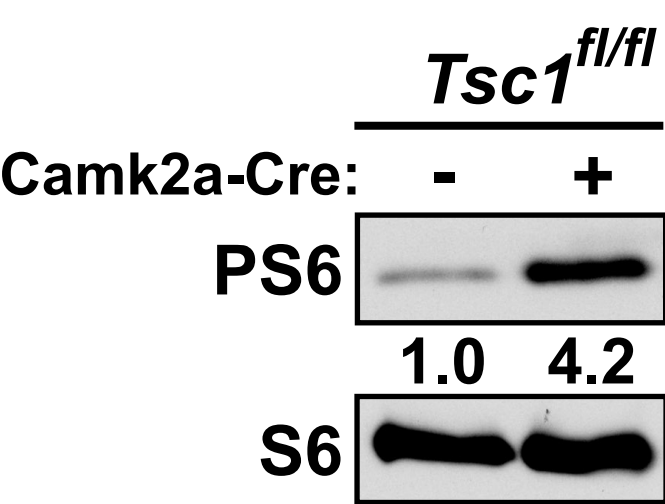

Figure panel

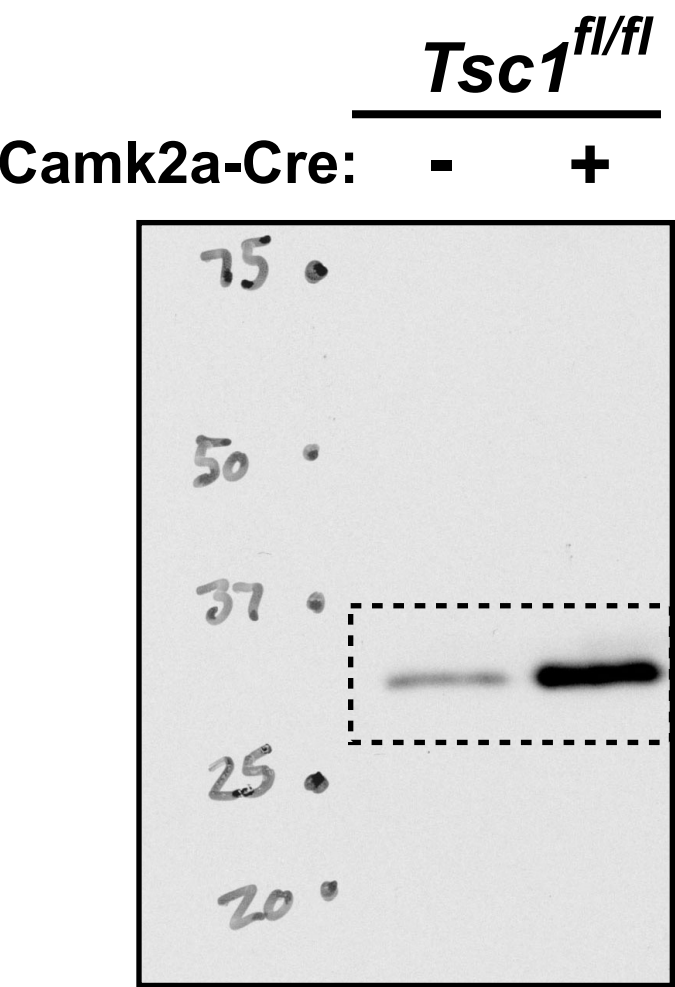

WB: PS6

Source data 2

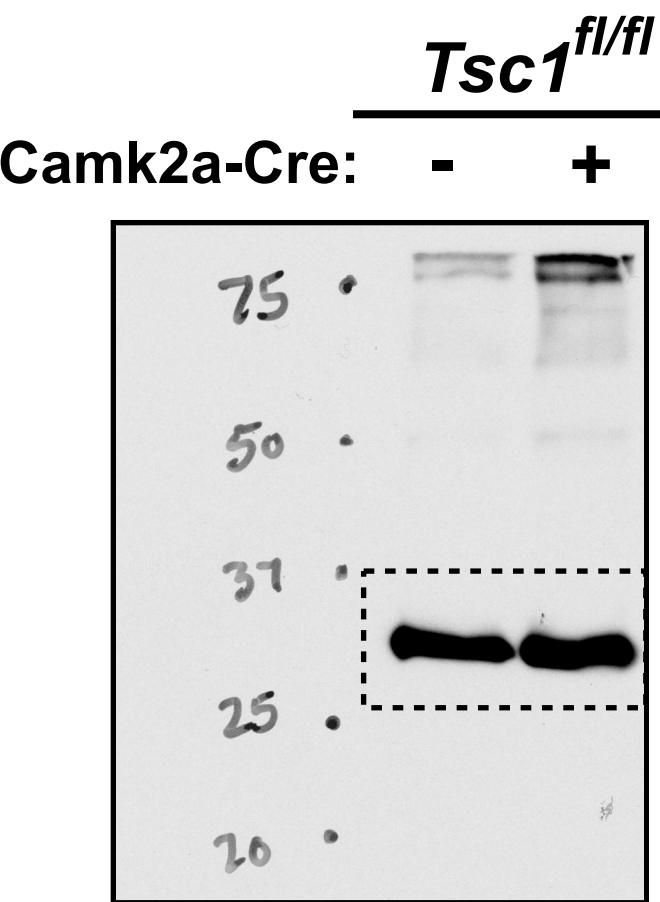

WB: S6

Source data 3

Supplement: Figure 1—figure supplement 1—source data 2. [file elife-85036-fig1-figsupp1-data2.zip › FigureS1C_sourcedata1.pdf]

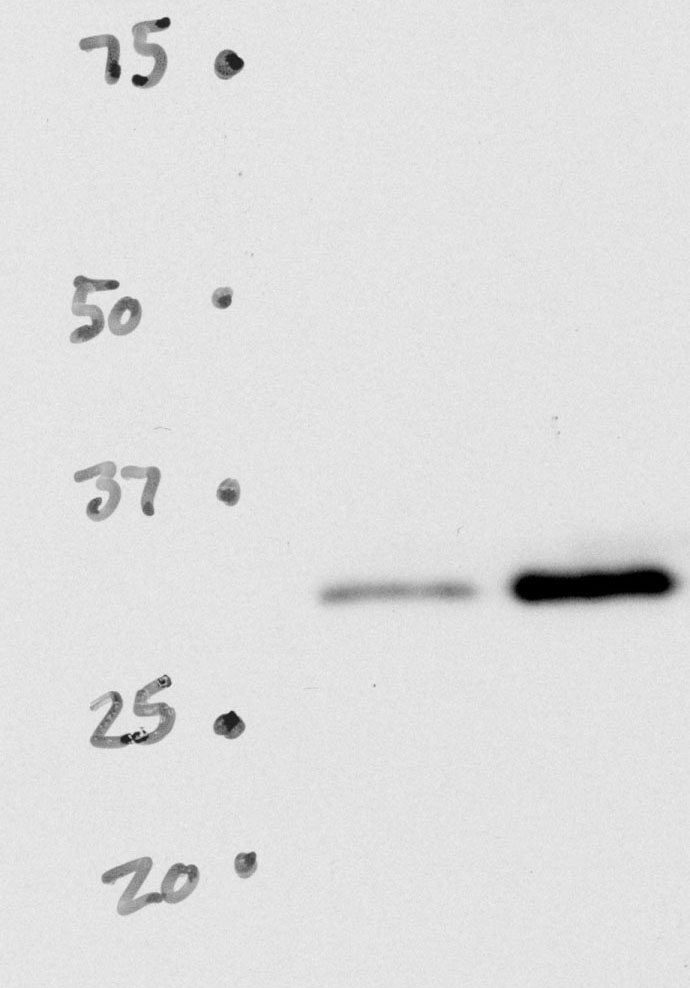

Supplement: Figure 1—figure supplement 1—source data 2. [file elife-85036-fig1-figsupp1-data2.zip › FigureS1C_sourcedata2.jpg]

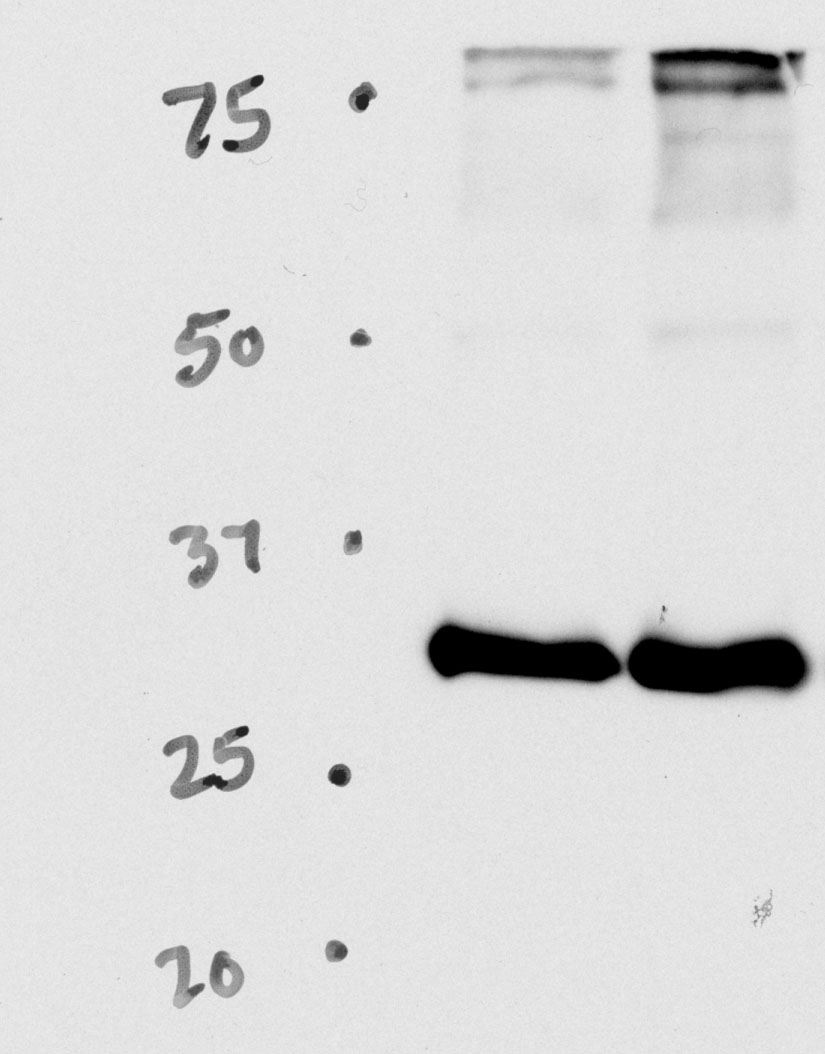

Supplement: Figure 1—figure supplement 1—source data 2. [file elife-85036-fig1-figsupp1-data2.zip › FigureS1C_sourcedata3.jpg]

Fig. 2A-source data

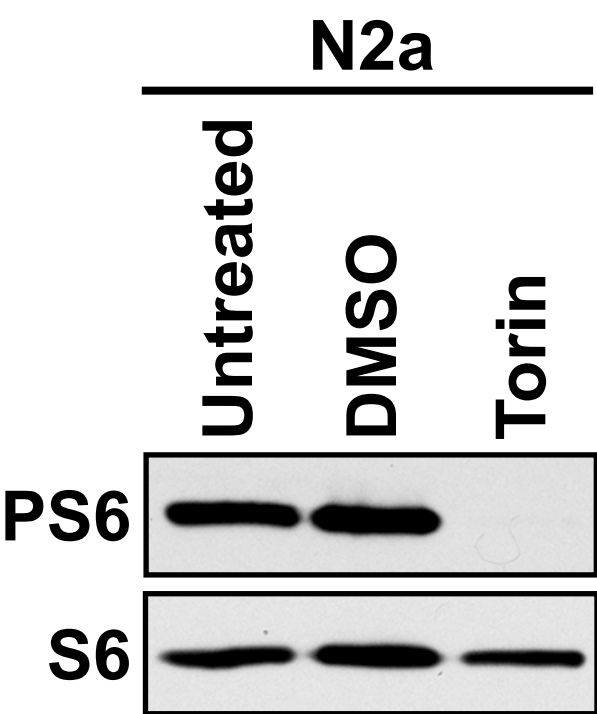

Figure panel

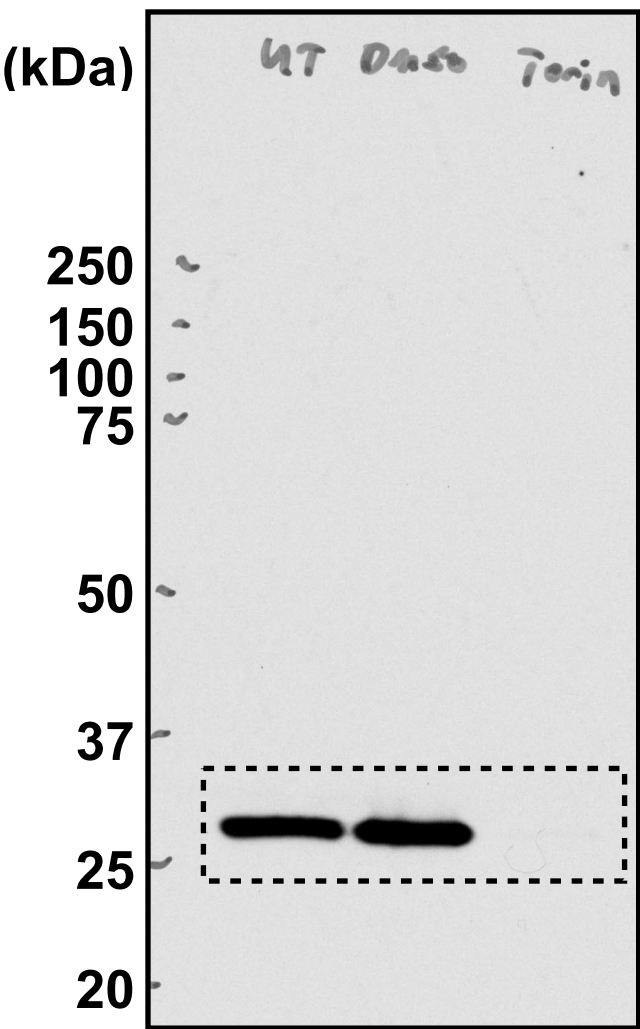

WB: PS6

Source data 2

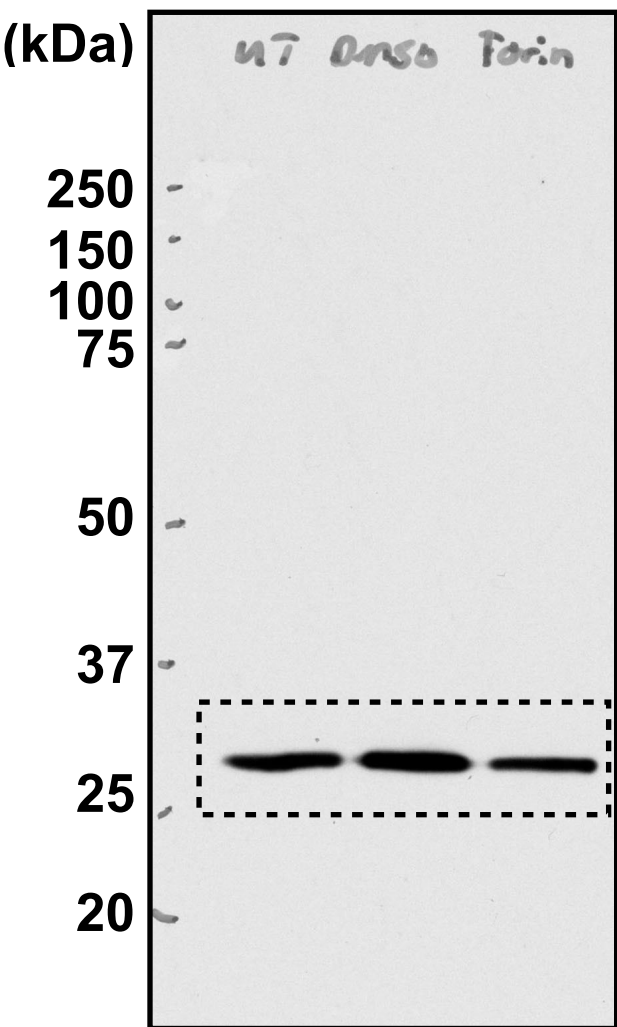

WB: S6

Source data 3

Supplement: Figure 2—source data 1. [file elife-85036-fig2-data1.zip › Figure2_source_data1/Figure2A_sourcedata1.pdf]

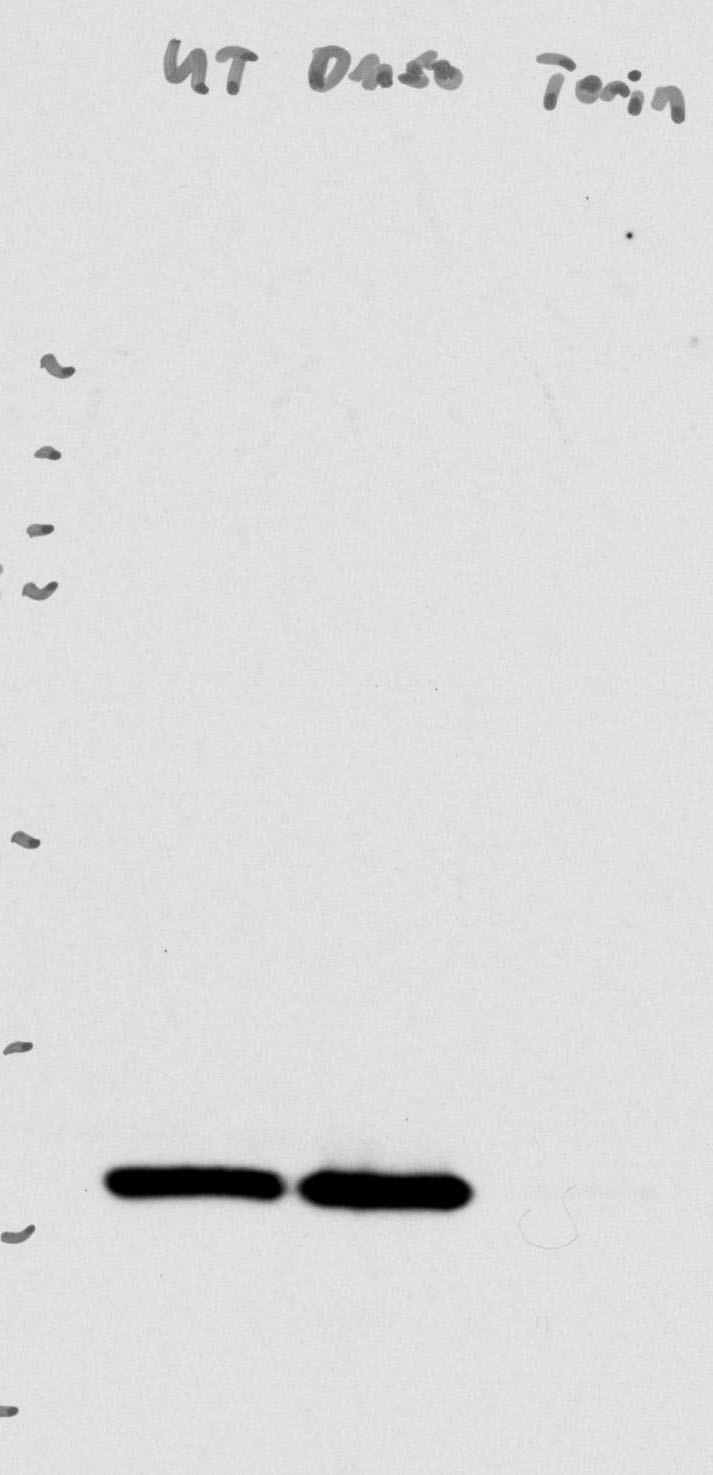

Supplement: Figure 2—source data 1. [file elife-85036-fig2-data1.zip › Figure2_source_data1/Figure2A_sourcedata2.jpg]

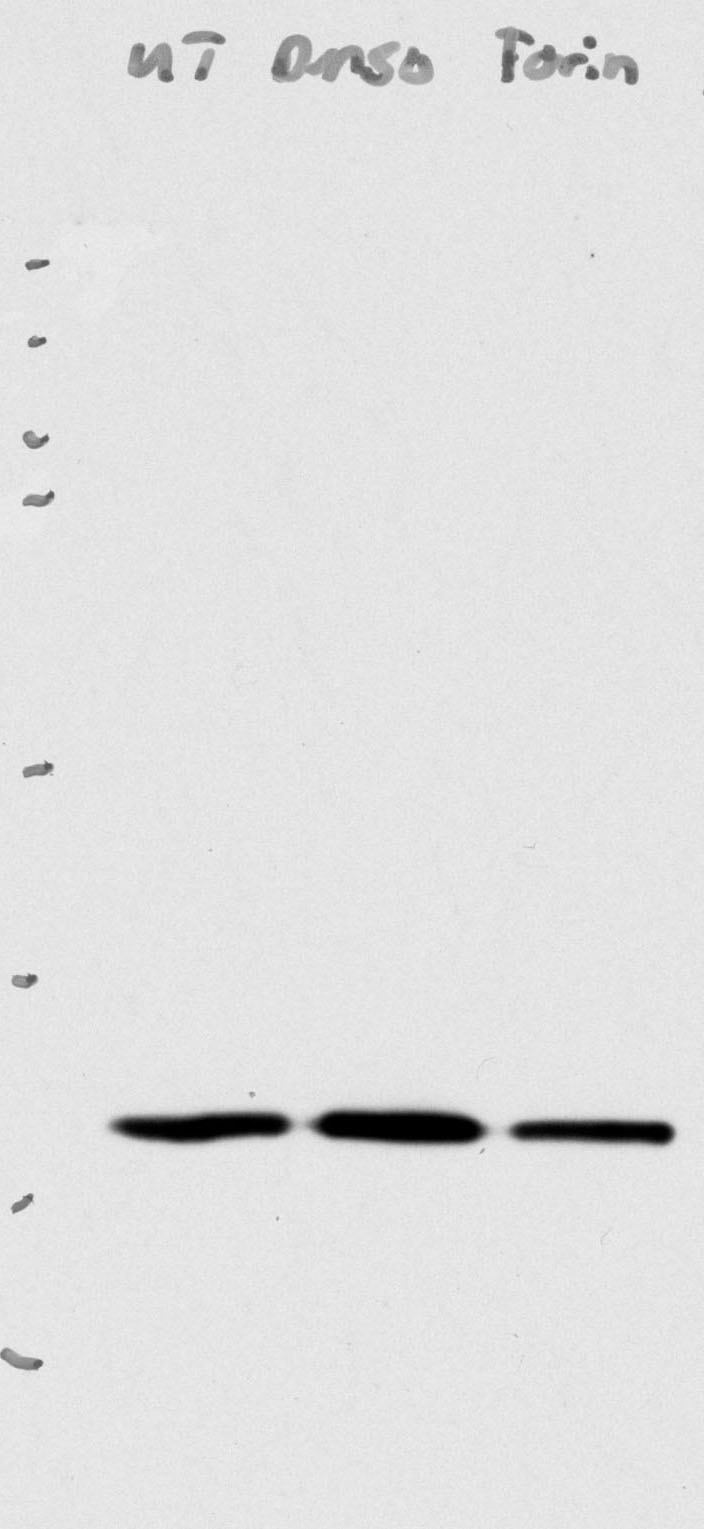

Supplement: Figure 2—source data 1. [file elife-85036-fig2-data1.zip › Figure2_source_data1/Figure2A_sourcedata3.jpg]

Fig. 2C-source data

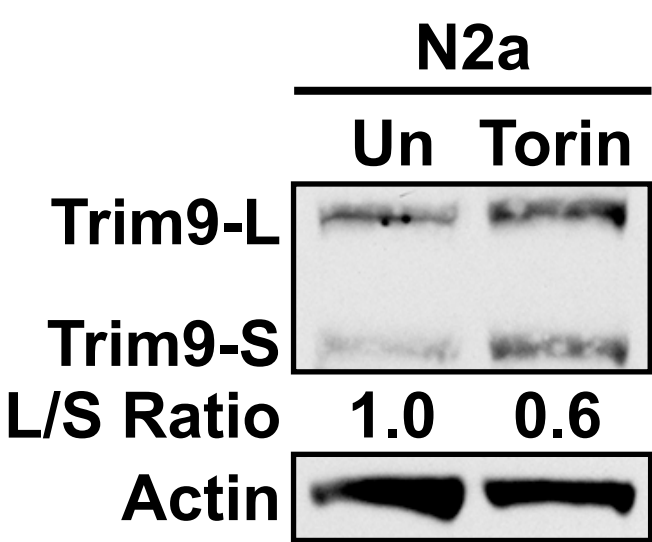

Figure panel

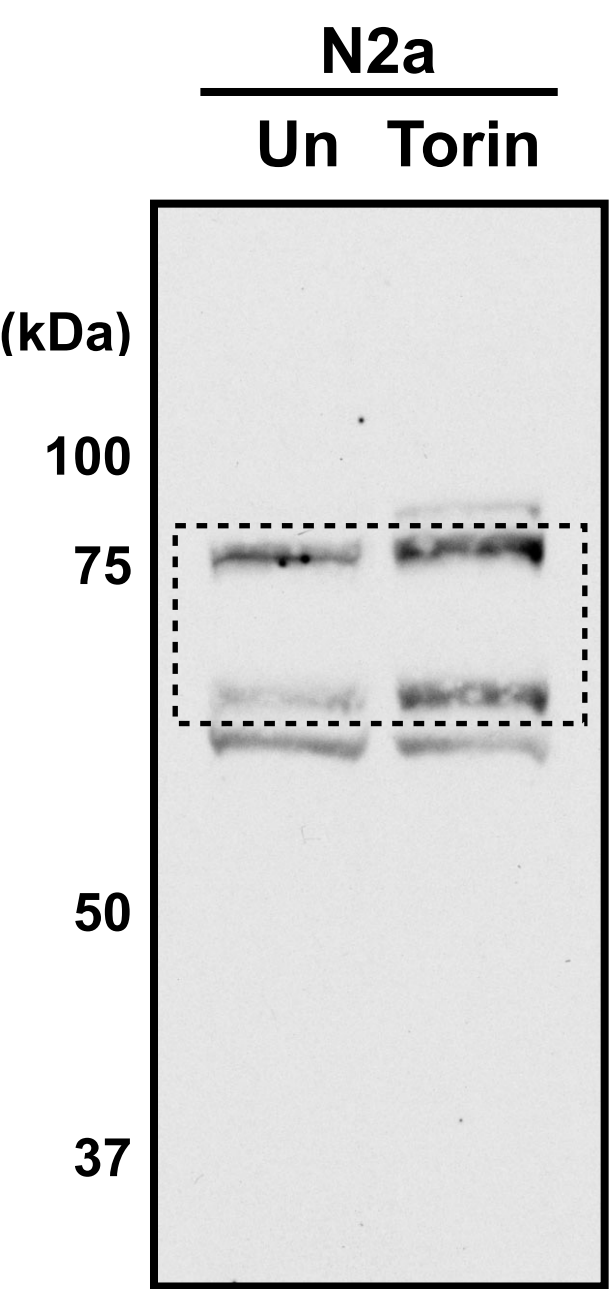

WB: Trim9

Source data 2

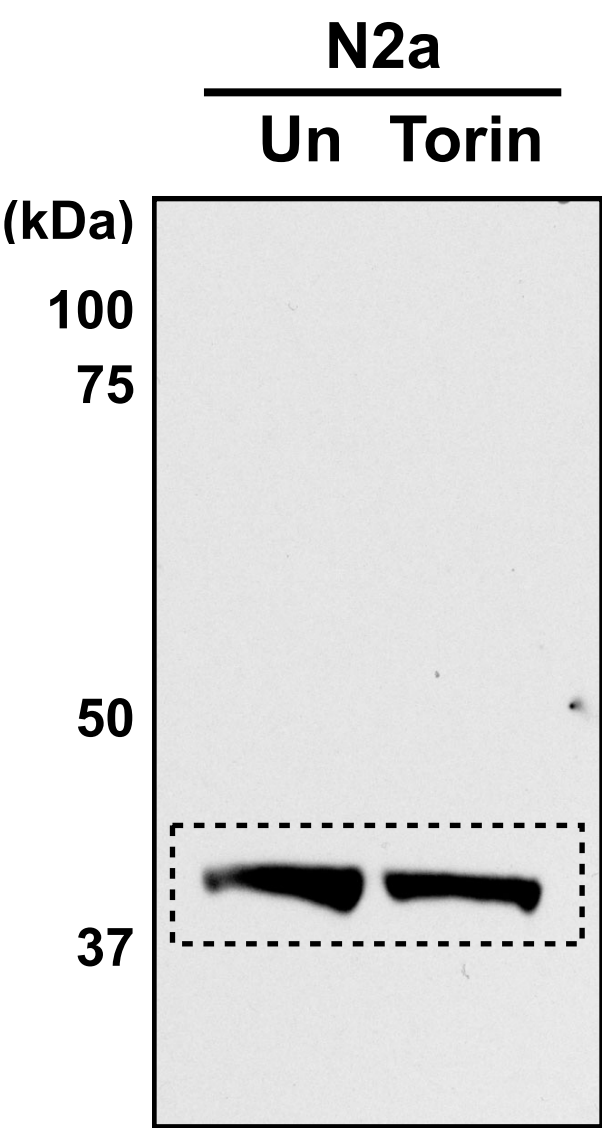

WB: Actin

Source data 3

Supplement: Figure 2—source data 2. [file elife-85036-fig2-data2.zip › Figure2_source_data2/Figure2C_sourcedata1.pdf]

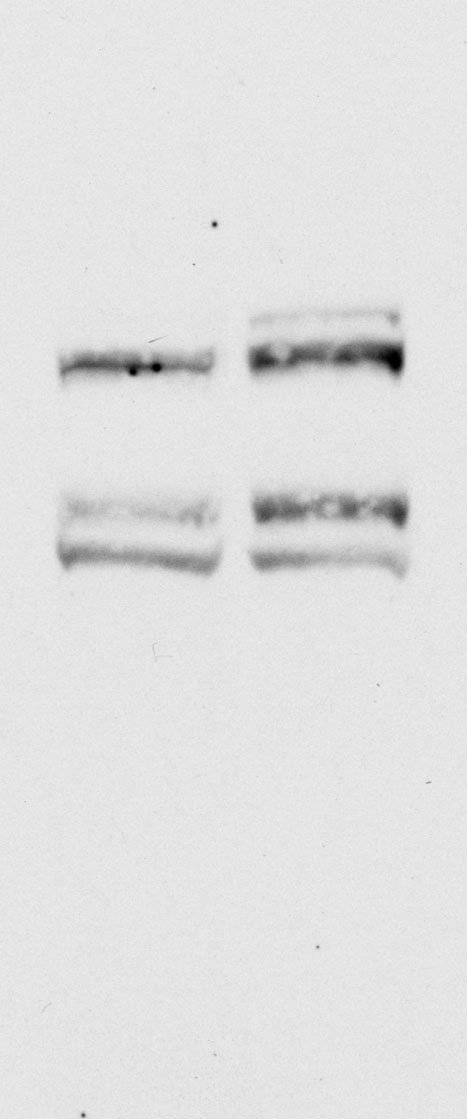

Supplement: Figure 2—source data 2. [file elife-85036-fig2-data2.zip › Figure2_source_data2/Figure2C_sourcedata2.jpg]

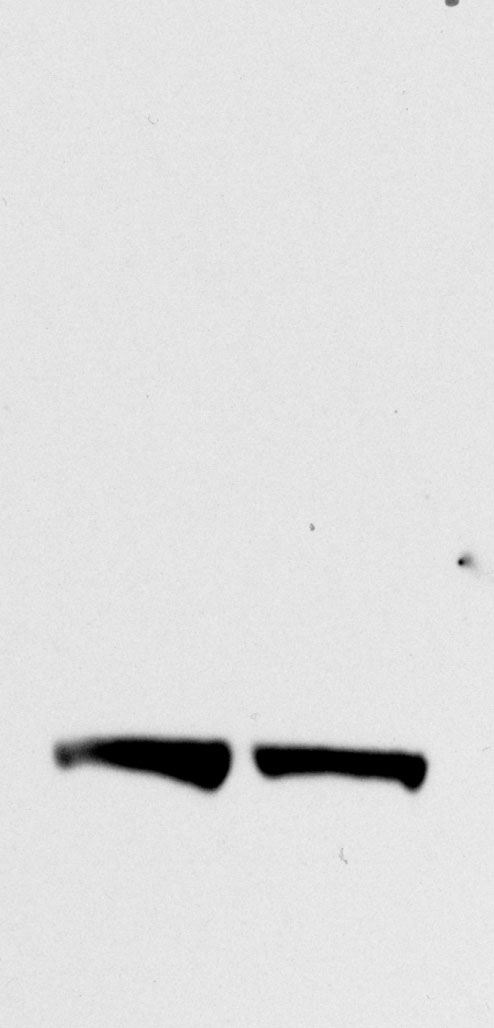

Supplement: Figure 2—source data 2. [file elife-85036-fig2-data2.zip › Figure2_source_data2/Figure2C_sourcedata3.jpg]

Fig. 2E-source data

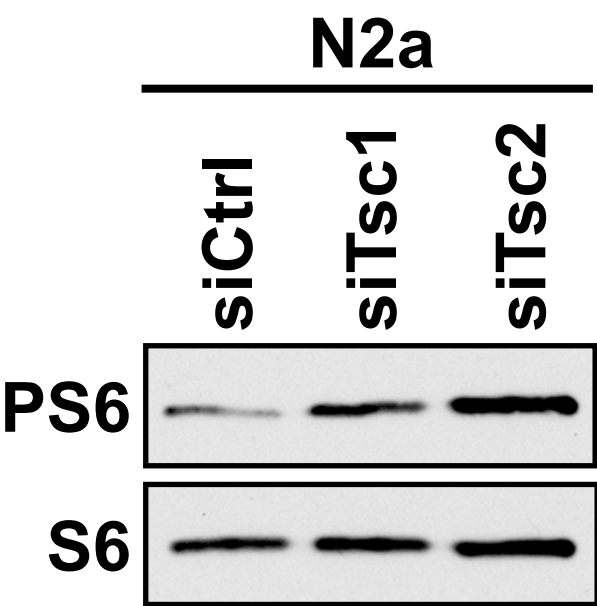

Figure panel

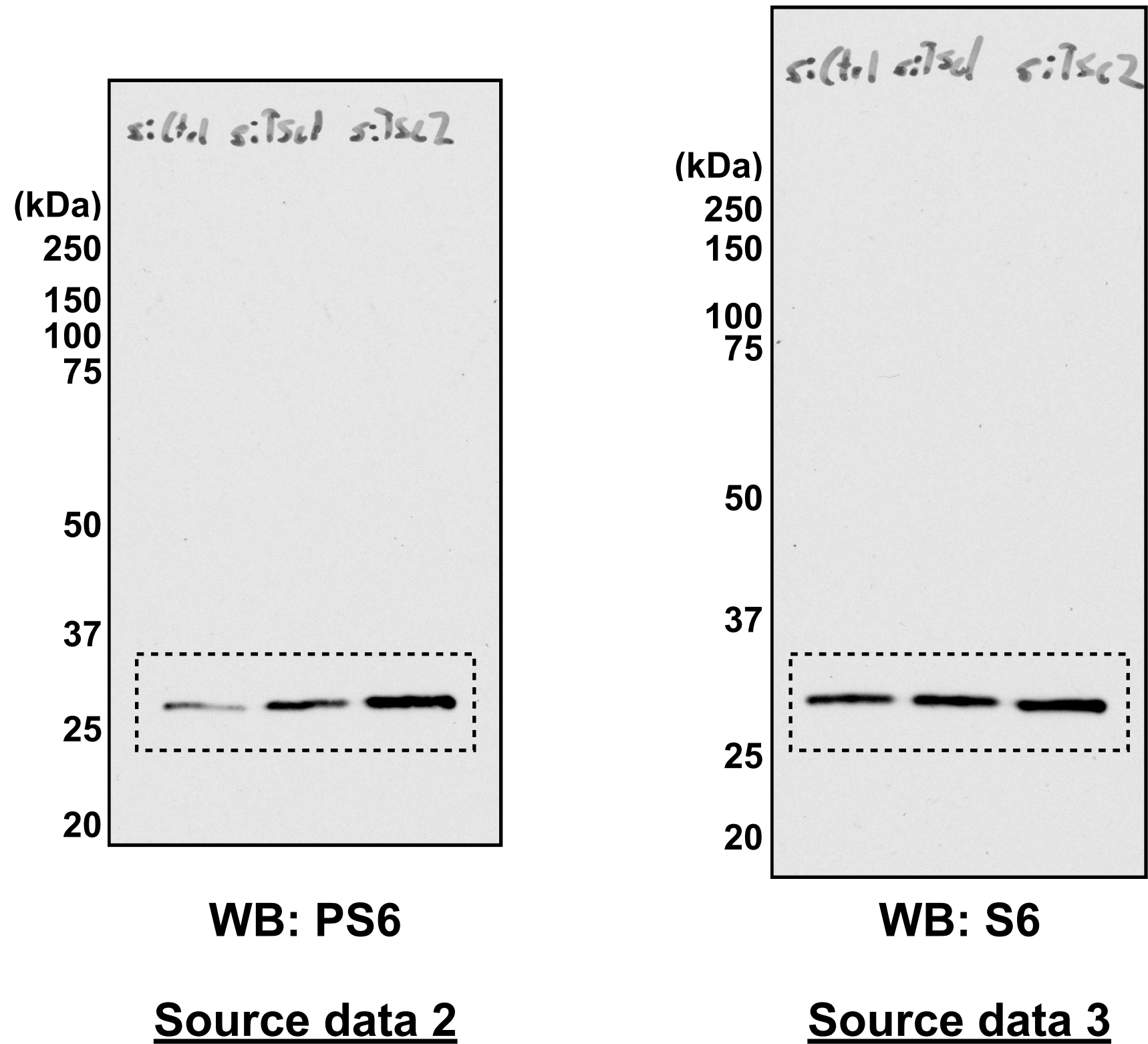

Supplement: Figure 2—source data 3. [file elife-85036-fig2-data3.zip › Figure2_source_data3/Figure2E_sourcedata1.pdf]

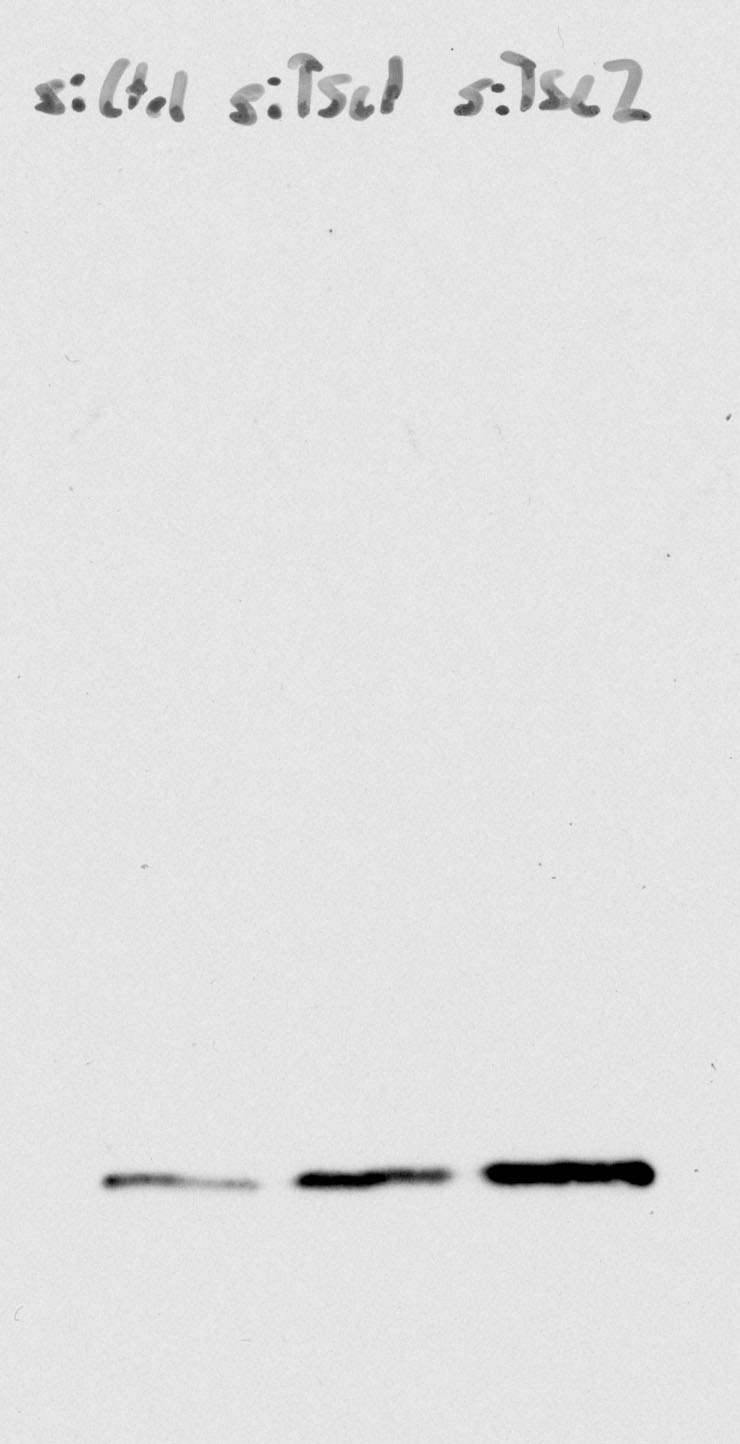

Supplement: Figure 2—source data 3. [file elife-85036-fig2-data3.zip › Figure2_source_data3/Figure2E_sourcedata2.jpg]

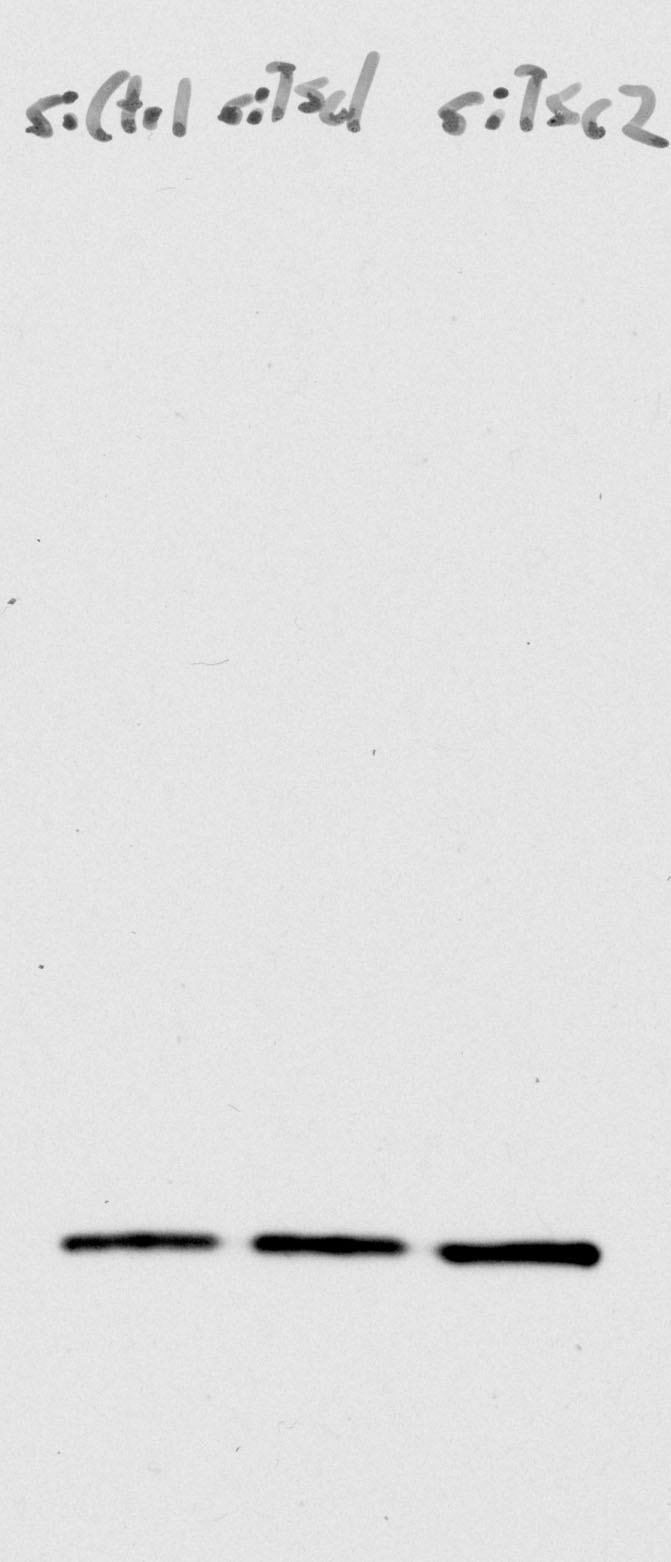

Supplement: Figure 2—source data 3. [file elife-85036-fig2-data3.zip › Figure2_source_data3/Figure2E_sourcedata3.jpg]

Fig. S2A-source data

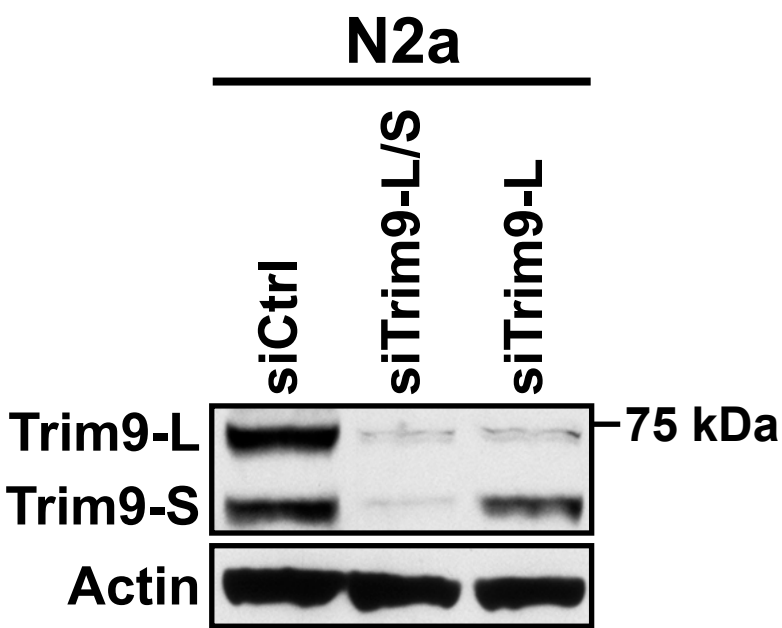

Figure panel

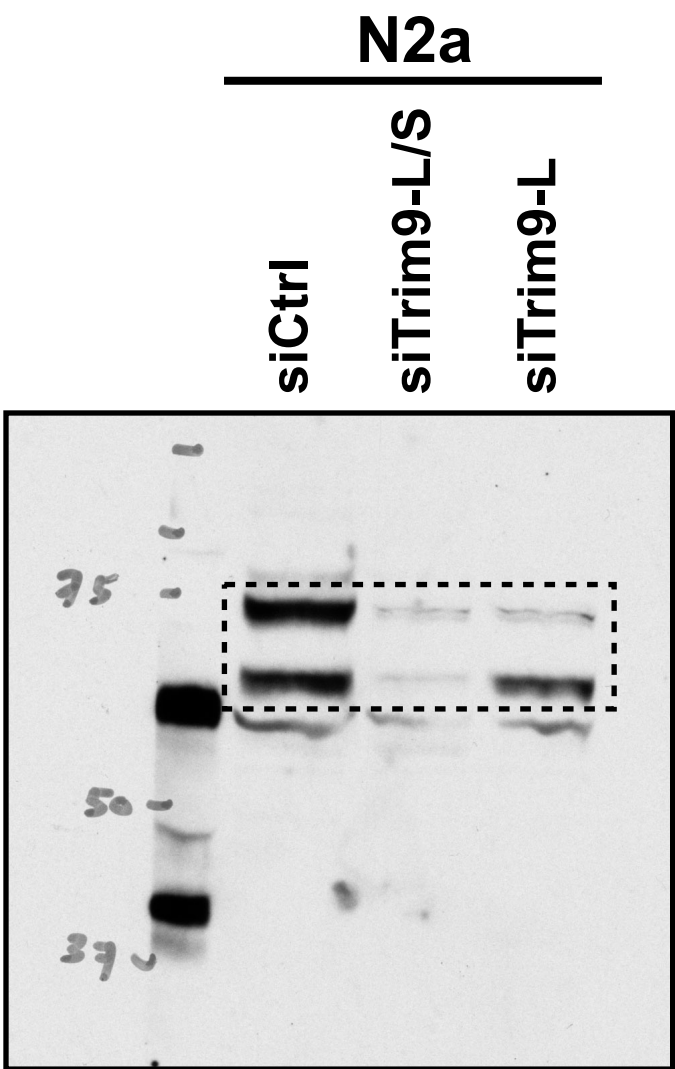

WB: Trim9

Source data 2

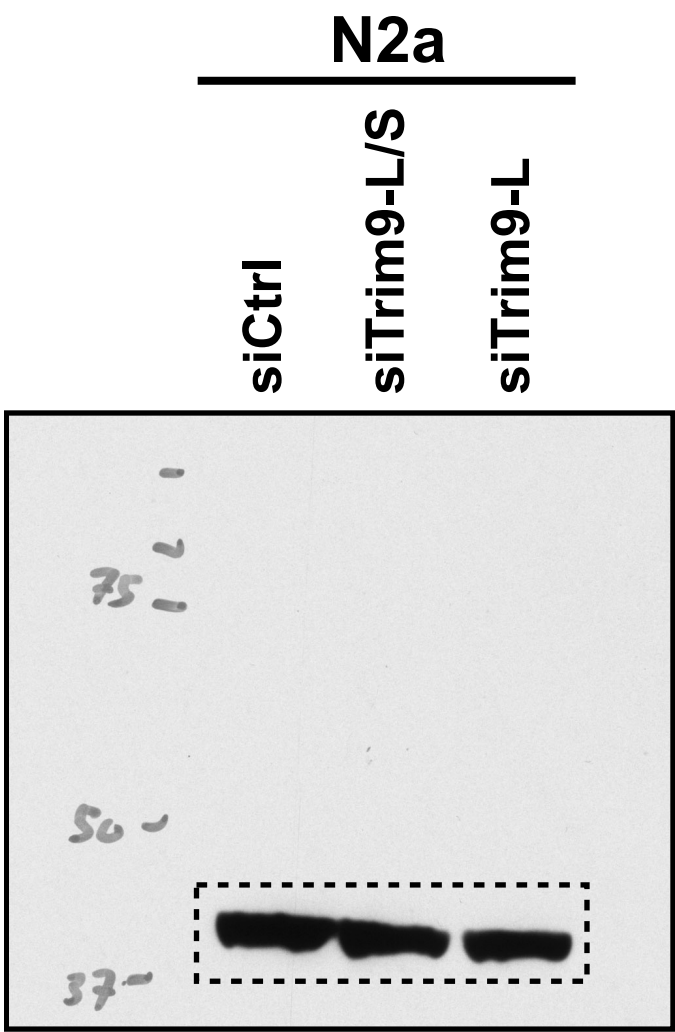

WB: Actin

Source data 3

Supplement: Figure 2—figure supplement 1—source data 1. [file elife-85036-fig2-figsupp1-data1.zip › Figure2_supplement1_source_data1/FigureS2A_sourcedata1.pdf]

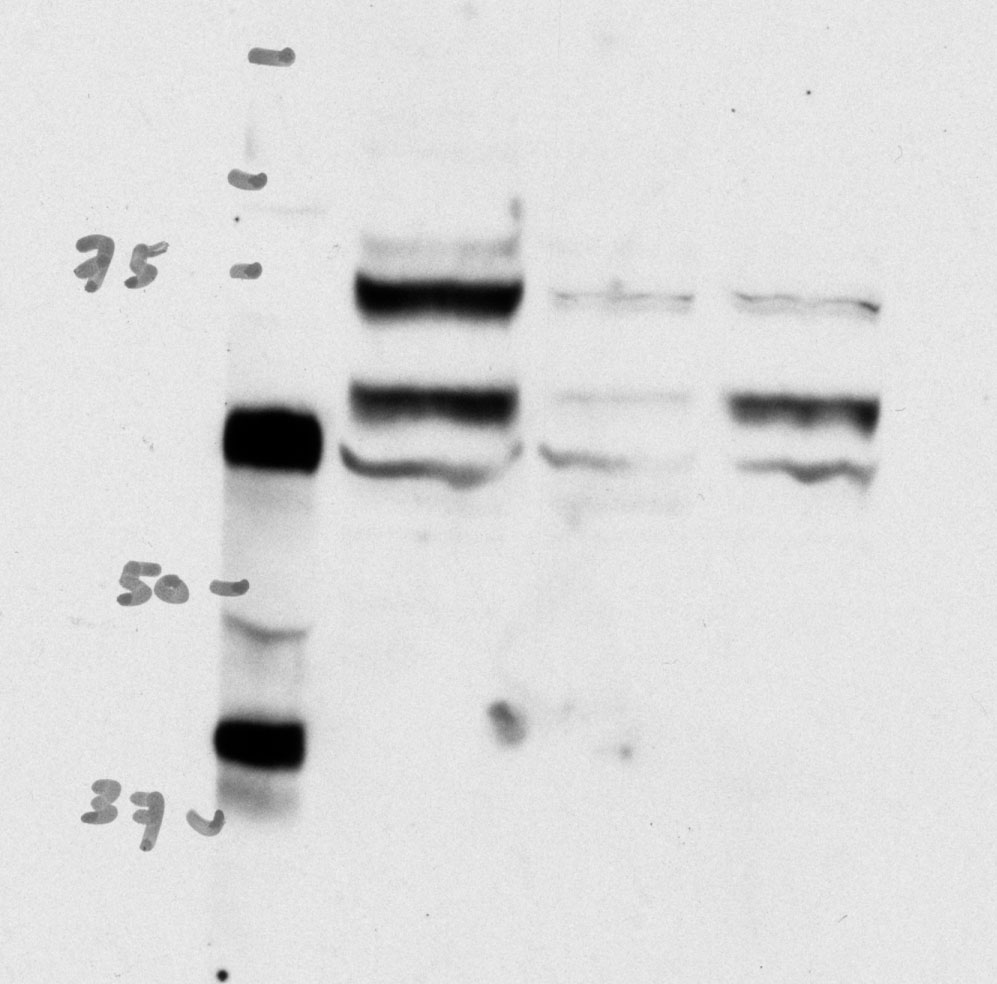

Supplement: Figure 2—figure supplement 1—source data 1. [file elife-85036-fig2-figsupp1-data1.zip › Figure2_supplement1_source_data1/FigureS2A_sourcedata2.jpg]

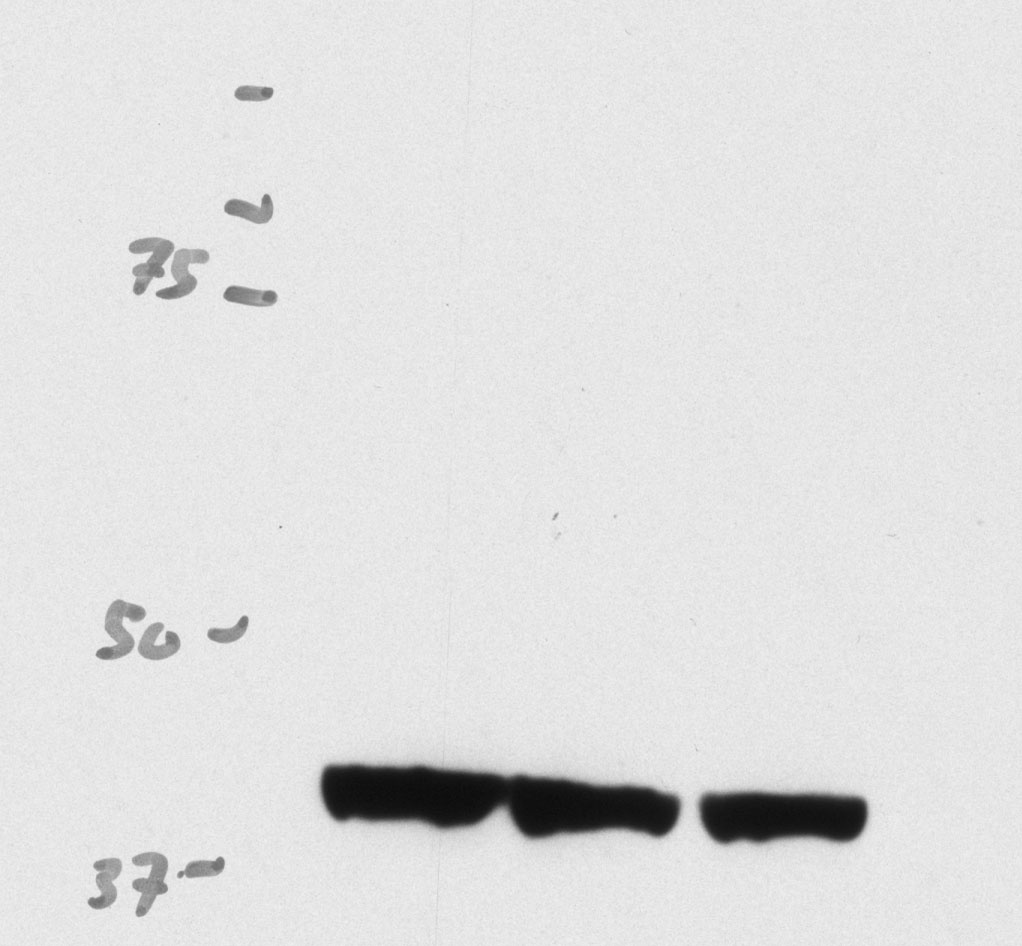

Supplement: Figure 2—figure supplement 1—source data 1. [file elife-85036-fig2-figsupp1-data1.zip › Figure2_supplement1_source_data1/FigureS2A_sourcedata3.jpg]

Fig. 3A-source data

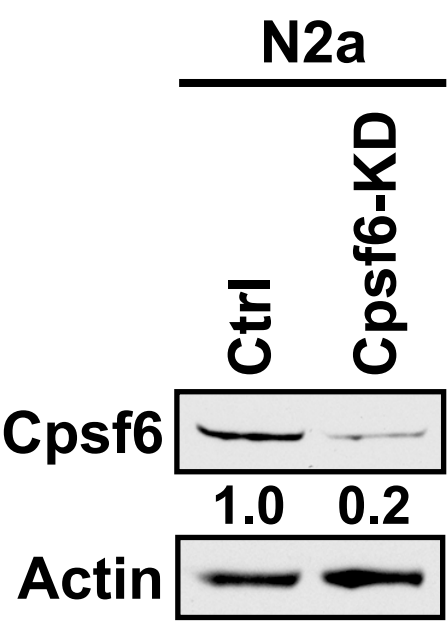

Figure panel

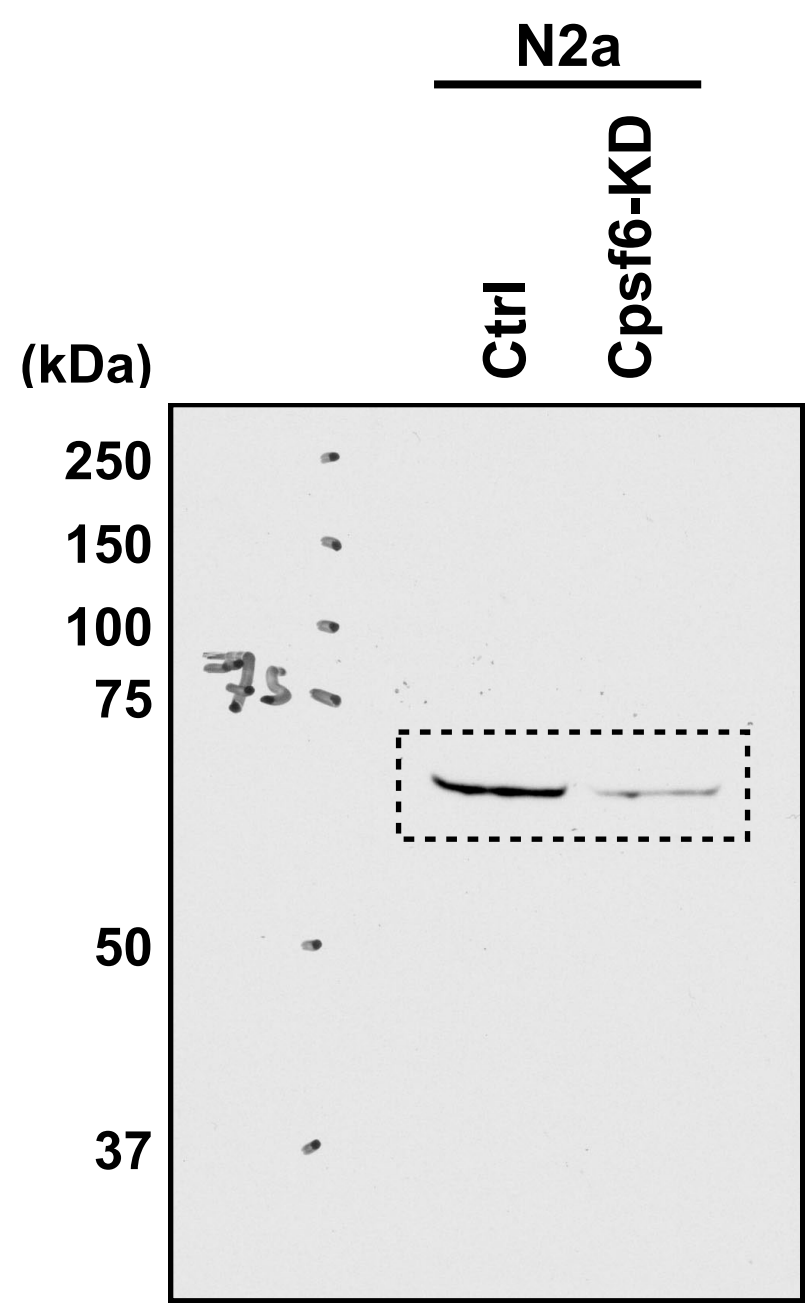

WB: CPSF6

Source data 2

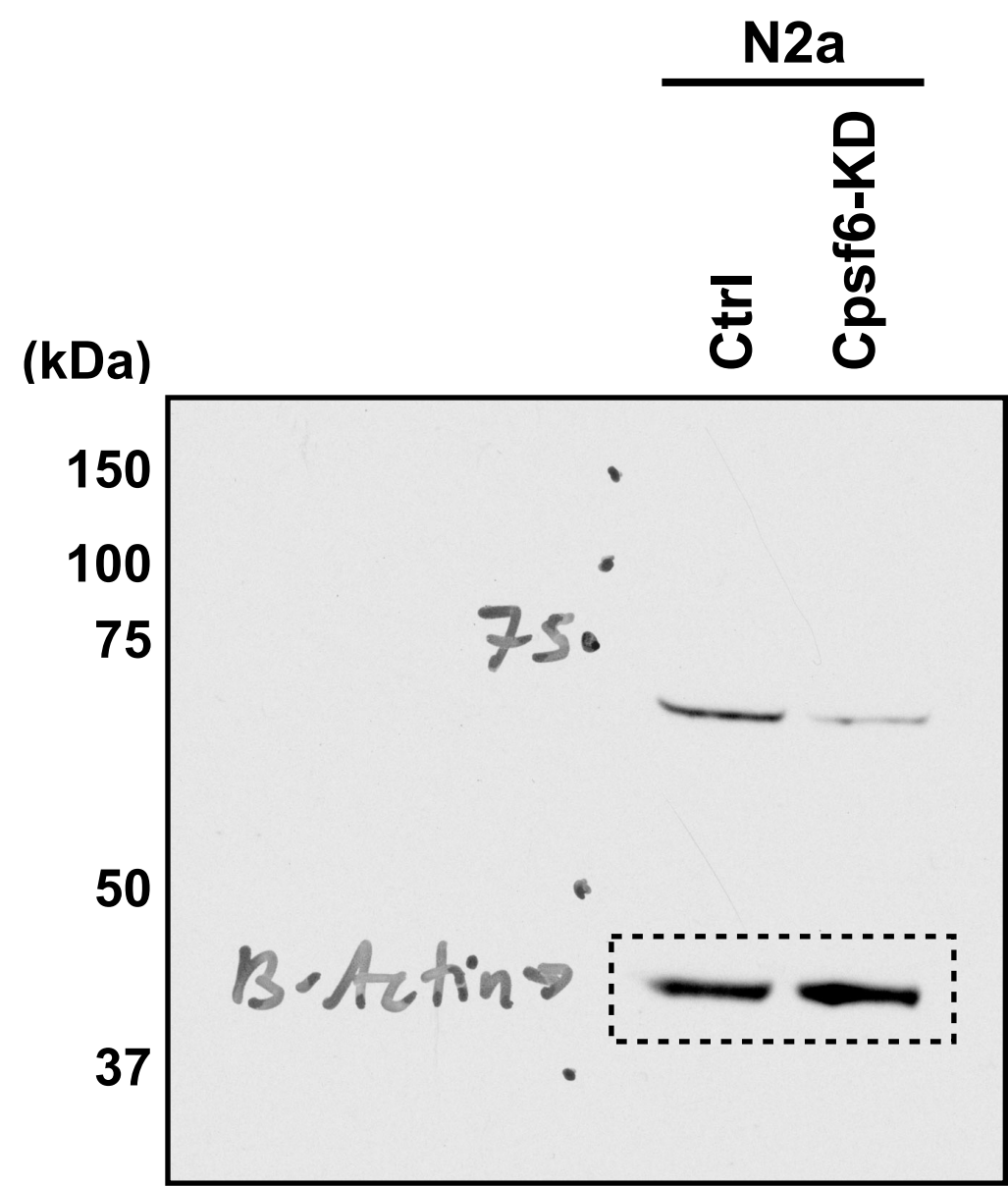

WB: Tubulin (re-blot)

Source data 3

Supplement: Figure 3—source data 1. [file elife-85036-fig3-data1.zip › Figure3_source_data1/Figure3A_sourcedata1.pdf]

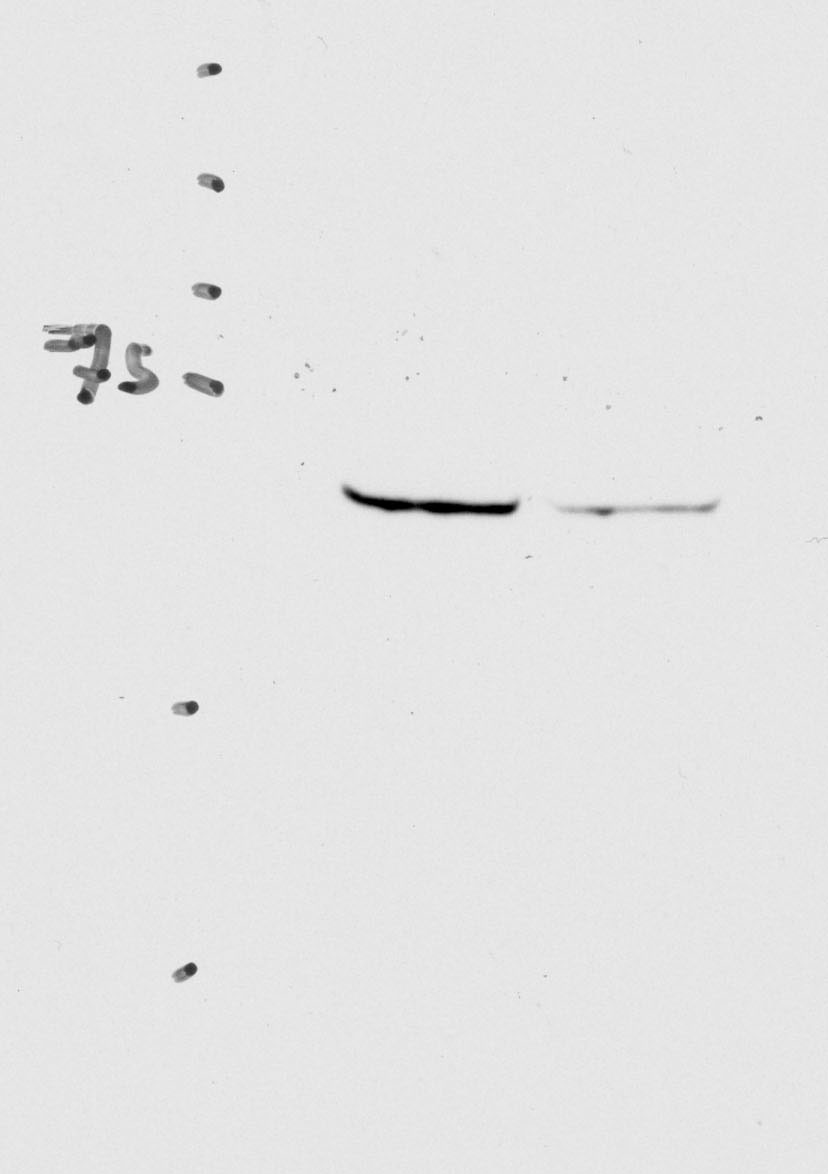

Supplement: Figure 3—source data 1. [file elife-85036-fig3-data1.zip › Figure3_source_data1/Figure3A_sourcedata2.jpg]

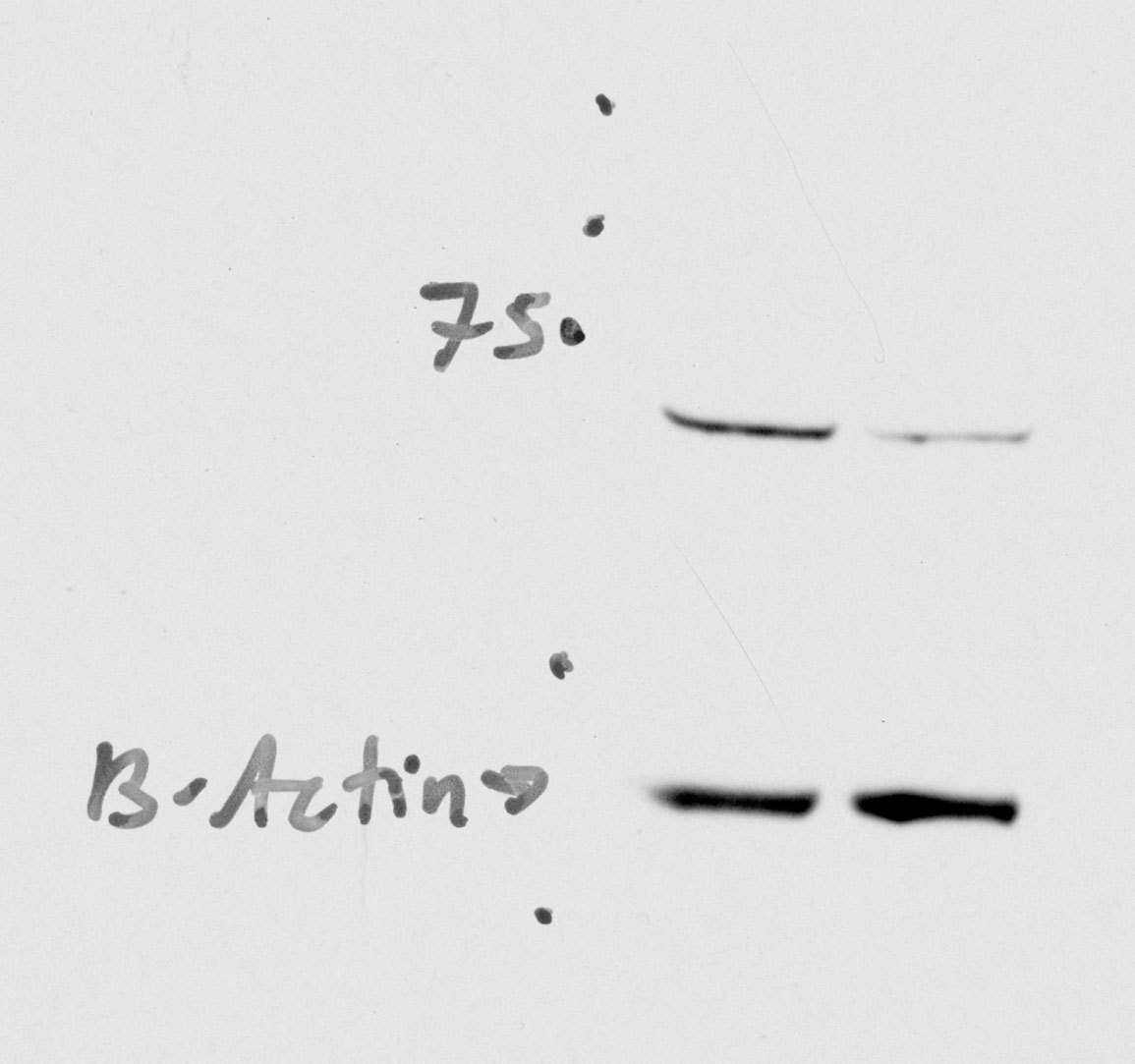

Supplement: Figure 3—source data 1. [file elife-85036-fig3-data1.zip › Figure3_source_data1/Figure3A_sourcedata3.jpg]

Fig. S4C-source data

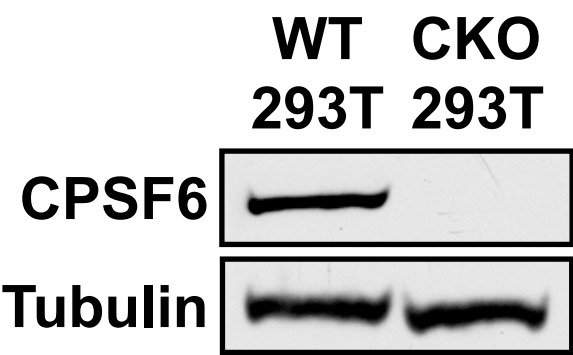

Figure panel

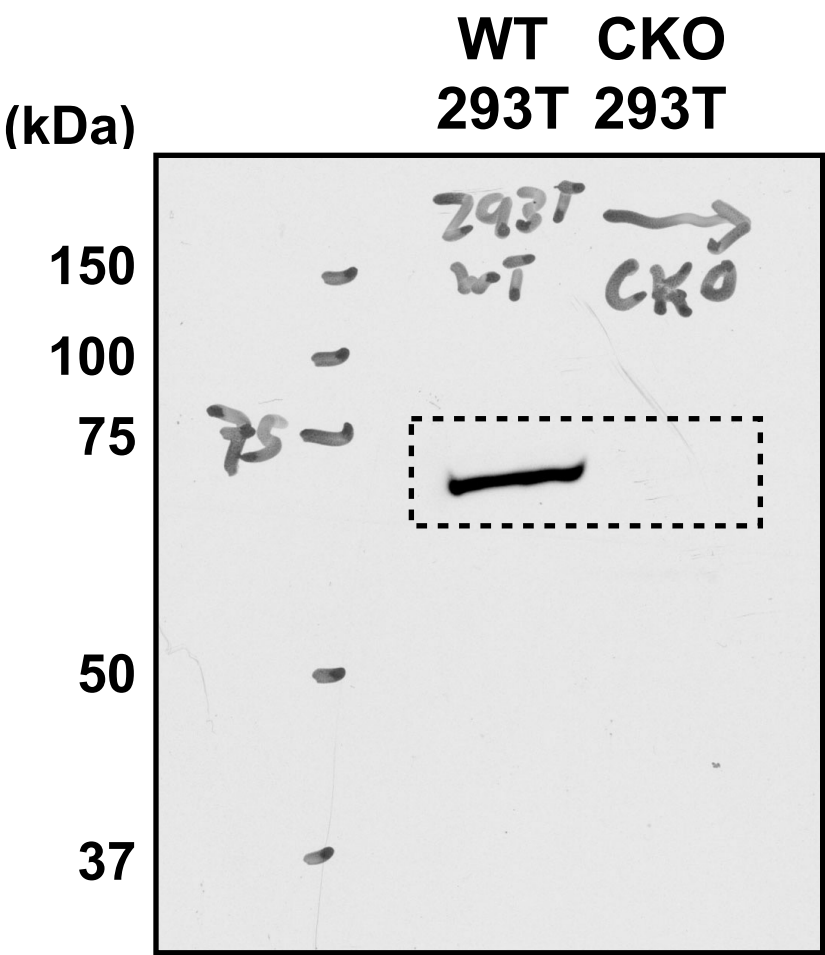

WB: CPSF6

Source data 2

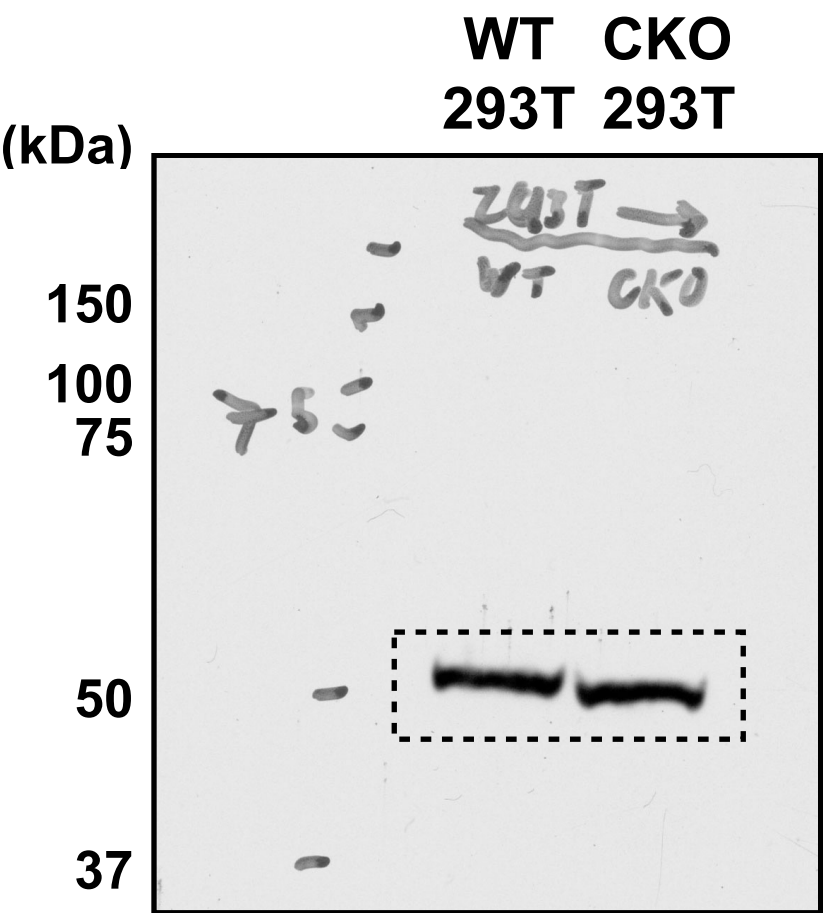

WB: Tubulin

Source data 3

Supplement: Figure 4—figure supplement 1—source data 1. [file elife-85036-fig4-figsupp1-data1.zip › Figure4_supplement1_source_data1/FigureS4C_sourcedata1.pdf]

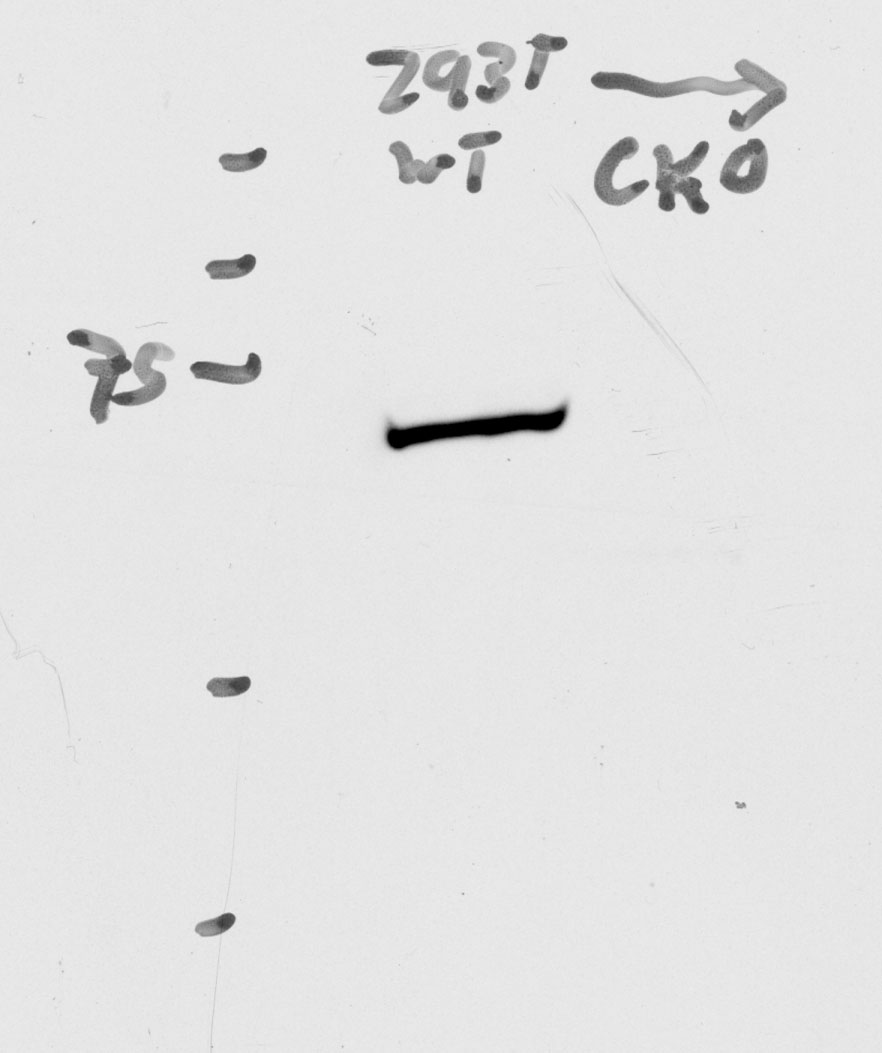

Supplement: Figure 4—figure supplement 1—source data 1. [file elife-85036-fig4-figsupp1-data1.zip › Figure4_supplement1_source_data1/FigureS4C_sourcedata2.jpg]

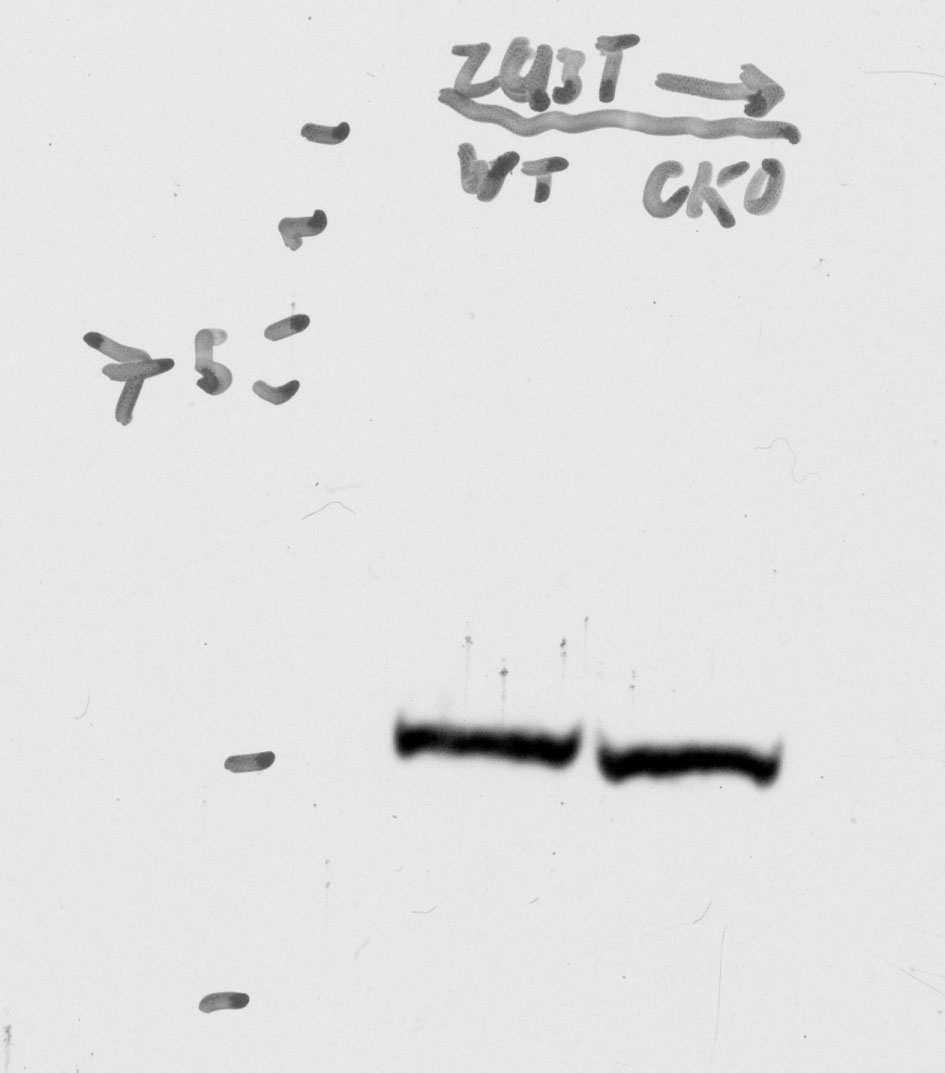

Supplement: Figure 4—figure supplement 1—source data 1. [file elife-85036-fig4-figsupp1-data1.zip › Figure4_supplement1_source_data1/FigureS4C_sourcedata3.jpg]

Fig. 5A-source data

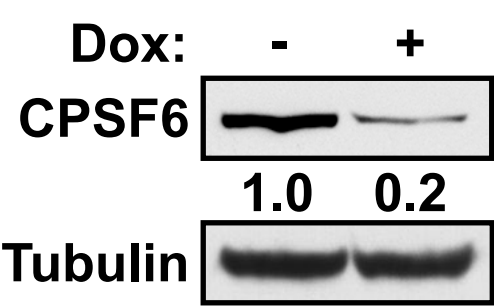

Figure panel

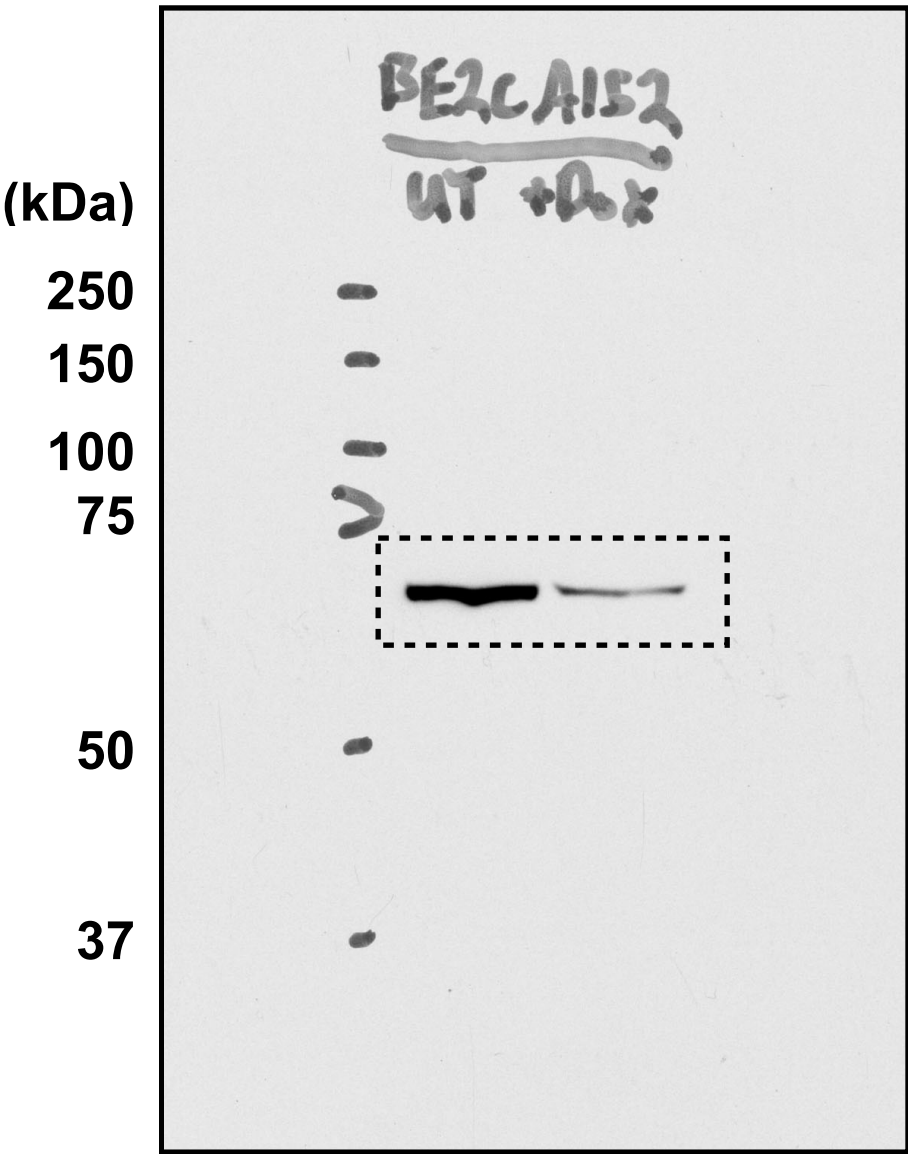

WB: CPSF6

Source data 2

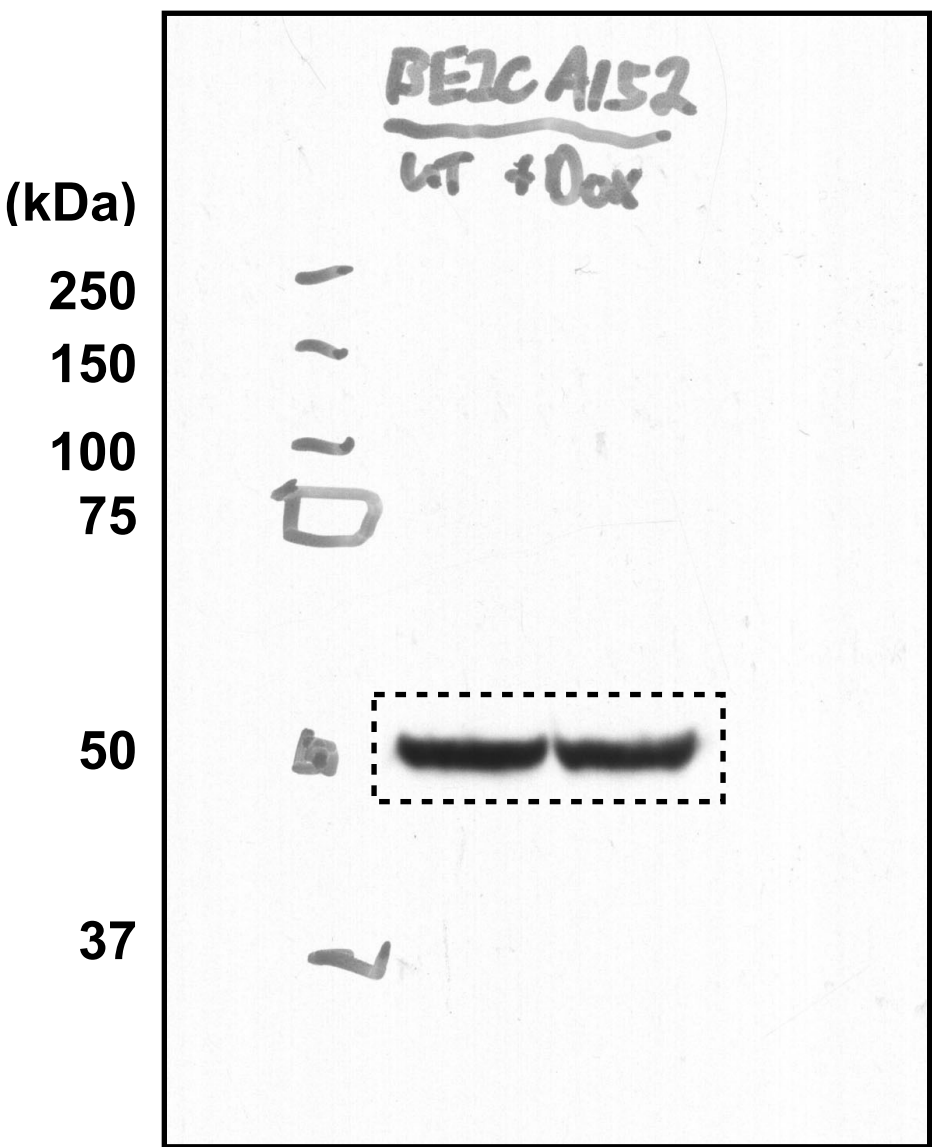

WB: Tubulin

Source data 3

Supplement: Figure 5—source data 1. [file elife-85036-fig5-data1.zip › Figure5_source_data1/Figure5A_sourcedata1.pdf]

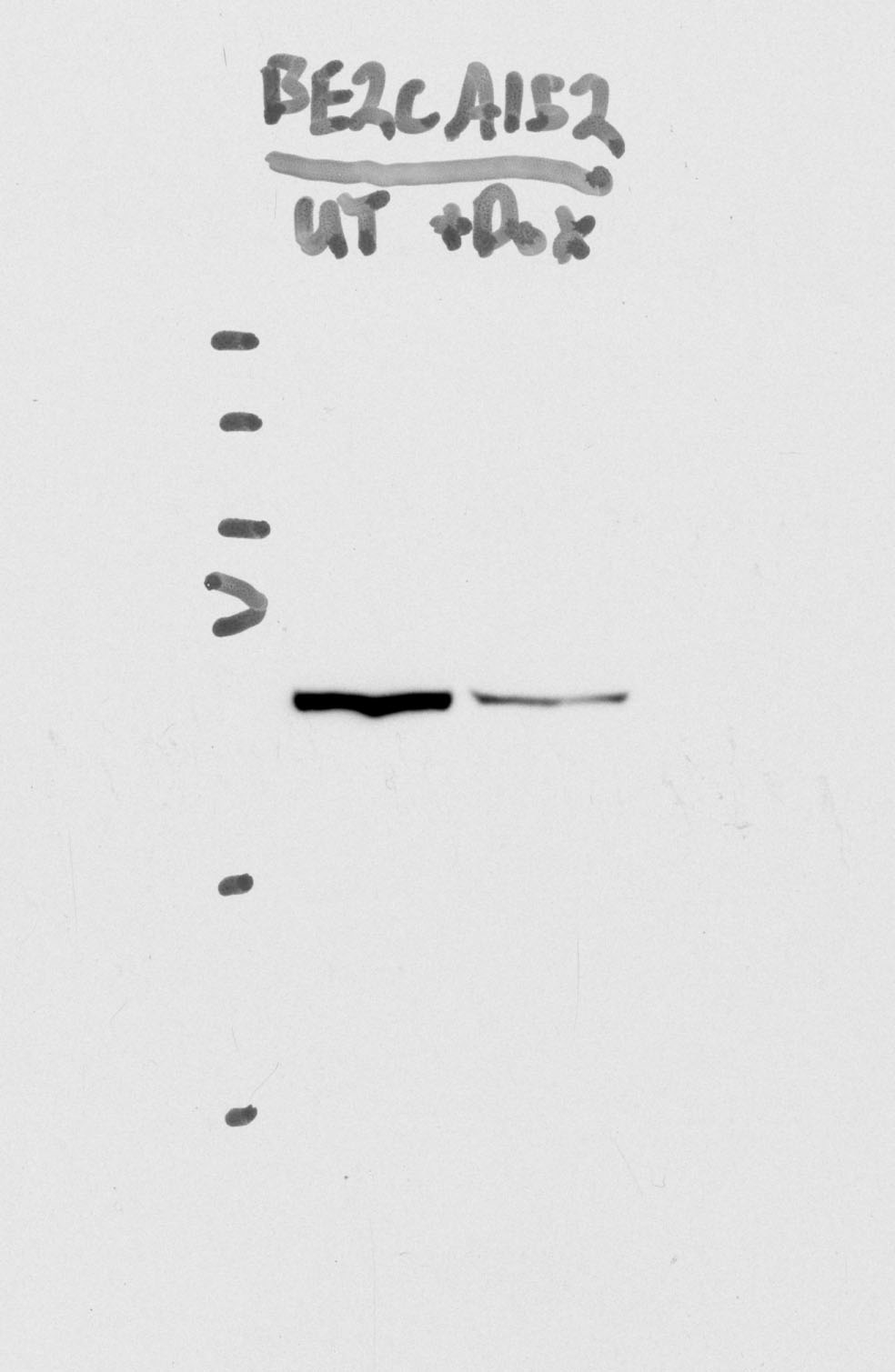

Supplement: Figure 5—source data 1. [file elife-85036-fig5-data1.zip › Figure5_source_data1/Figure5A_sourcedata2.jpg]

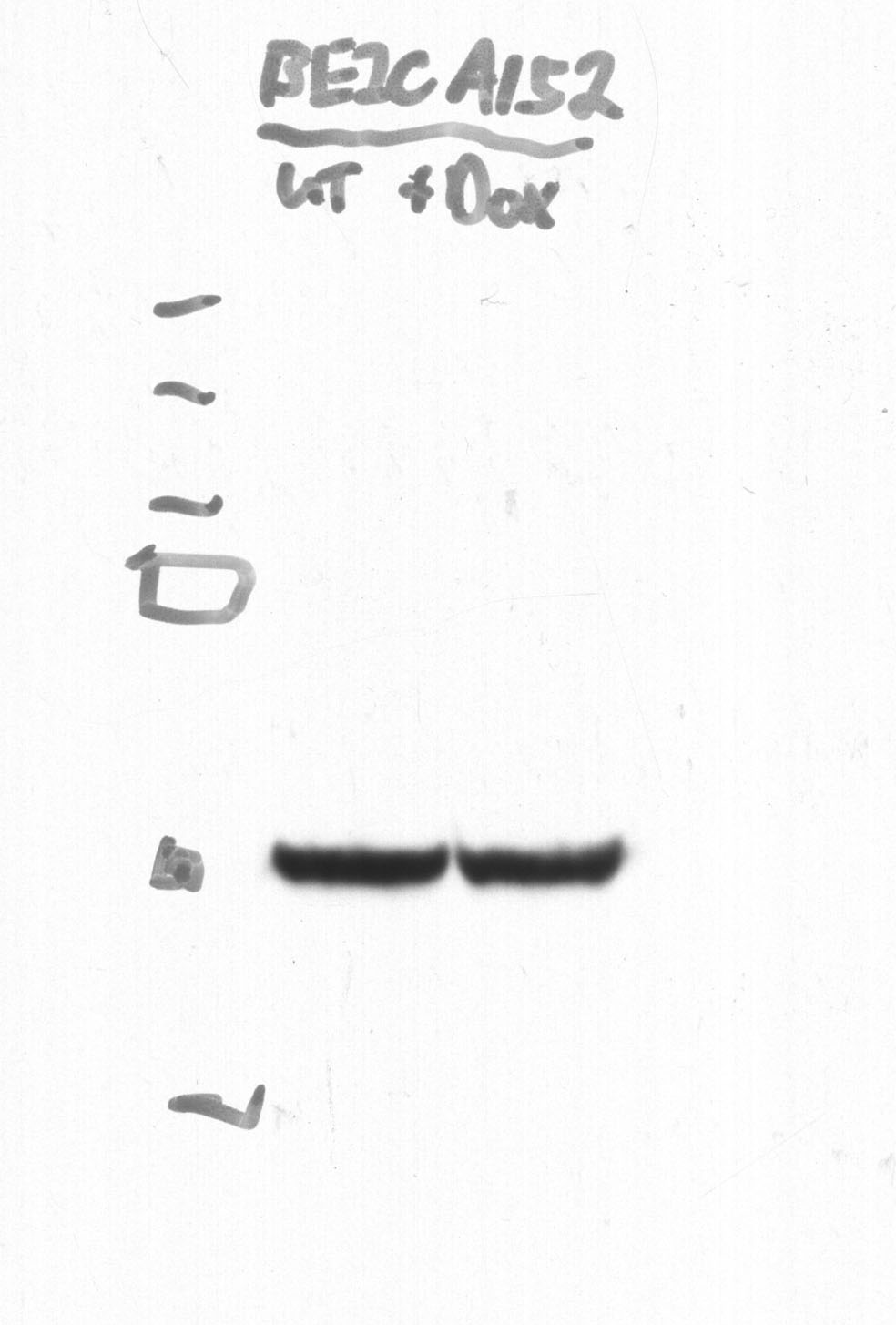

Supplement: Figure 5—source data 1. [file elife-85036-fig5-data1.zip › Figure5_source_data1/Figure5A_sourcedata3.jpg]

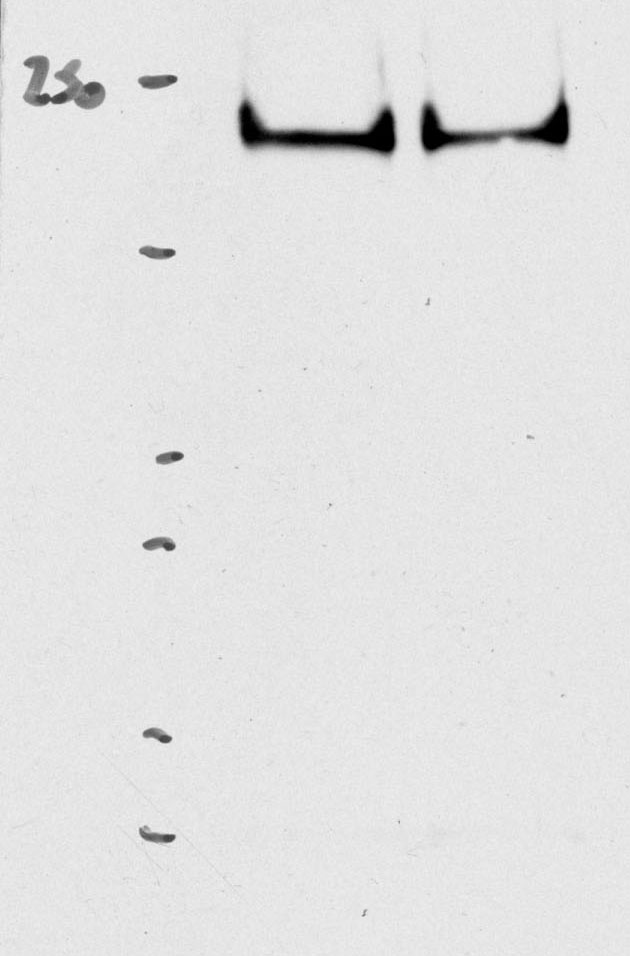

Supplement: Figure 6—source data 1. [file elife-85036-fig6-data1.zip › Figure6_source_data1/Figure6D_sourcedata2.jpg]

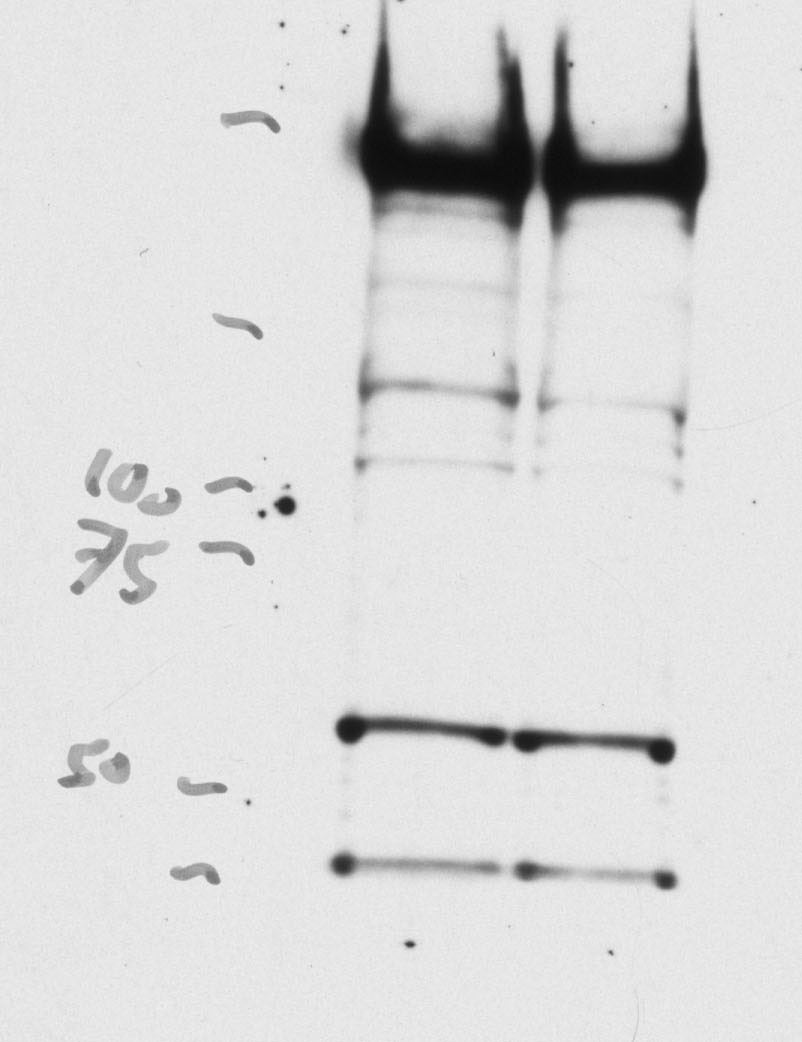

Supplement: Figure 6—source data 1. [file elife-85036-fig6-data1.zip › Figure6_source_data1/Figure6D_sourcedata3.jpg]

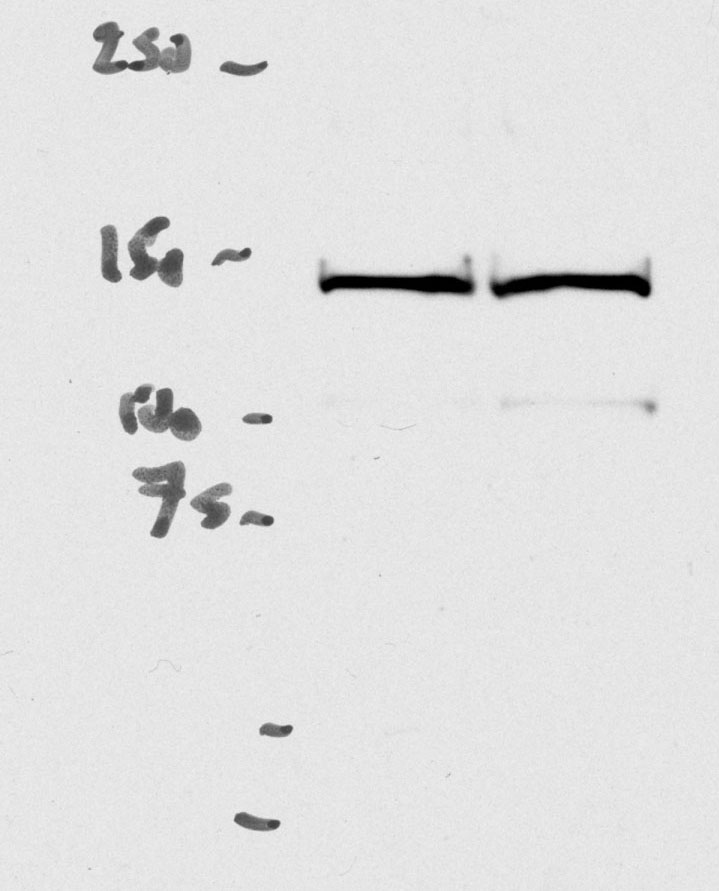

Supplement: Figure 6—source data 1. [file elife-85036-fig6-data1.zip › Figure6_source_data1/Figure6D_sourcedata4.jpg]

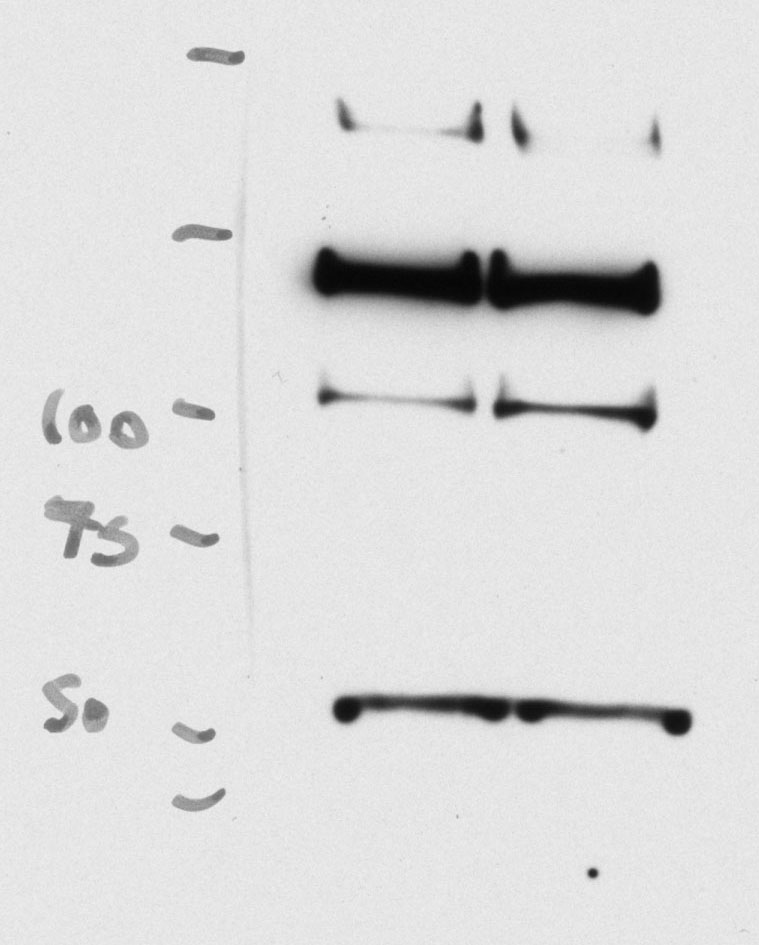

Supplement: Figure 6—source data 1. [file elife-85036-fig6-data1.zip › Figure6_source_data1/Figure6D_sourcedata5.jpg]
