# Supplementary material for: A twin UGUA motif directs the balance between gene isoforms through CFIm and the mTORC1 signaling pathway
Source: eLife. 2023 Sep 4;12:e85036. doi: 10.7554/eLife.85036 (PMC10476966; doi:10.7554/eLife.85036)
Supplement: Figure 6—source data 1. [file elife-85036-fig6-data1.zip › Figure6_source_data1/Figure6D_sourcedata1.pdf]

Fig. 6D-source data

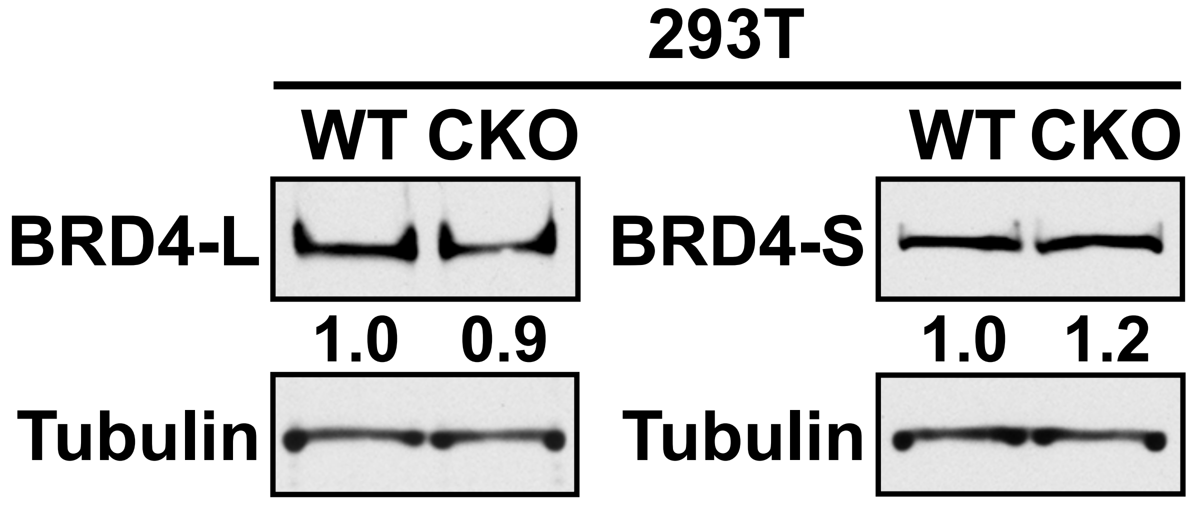

Figure panel

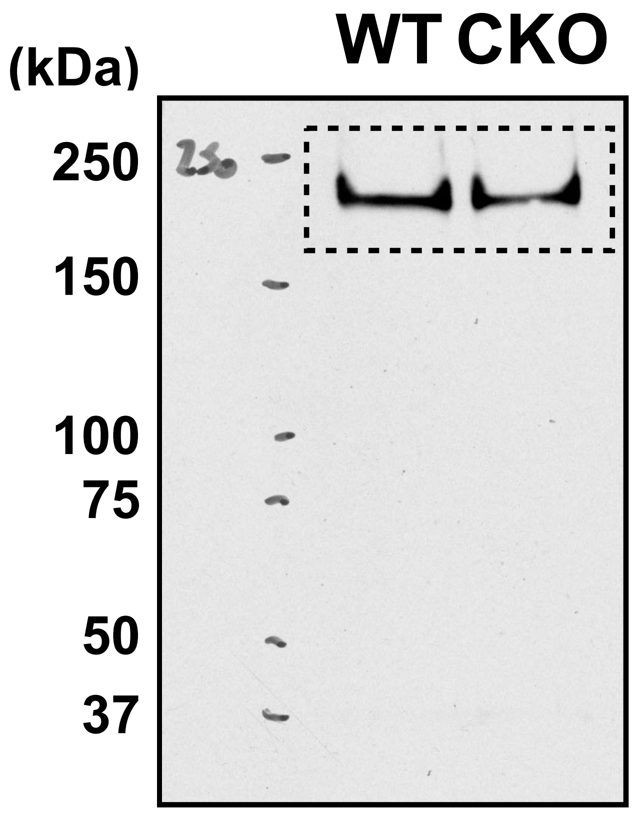

WB: BRD4-L

Source data 2

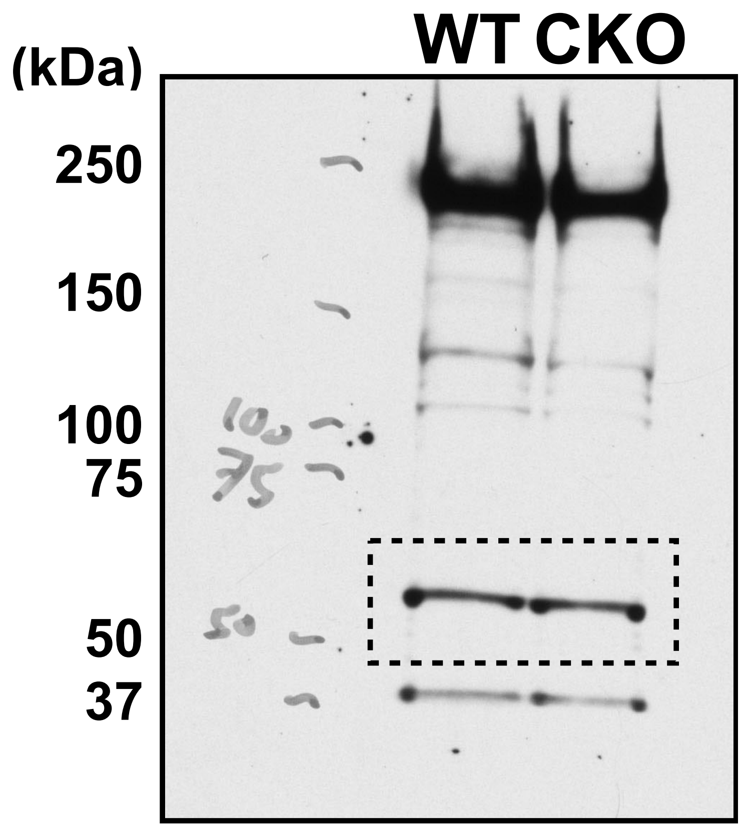

WB: Tubulin (re-blot)

Source data 3

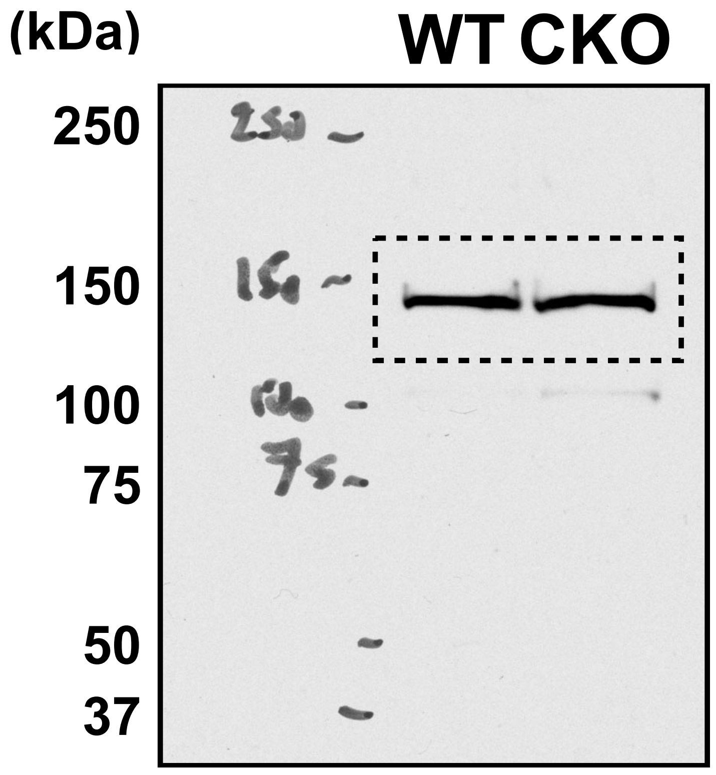

WB: BRD4-S

Source data 4

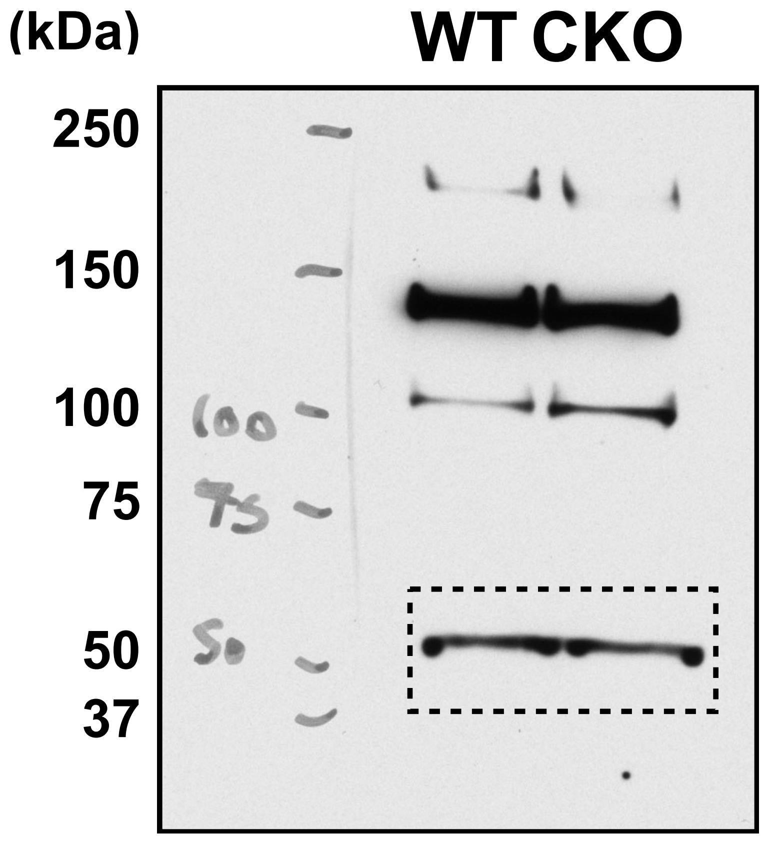

WB: Tubulin (re-blot)

Source data 5
